# Strategies for preventing gender‐based violence in healthcare services: Evidence synthesis for health policy

**DOI:** 10.1002/ijgo.70934

**Published:** 2026-03-23

**Authors:** Odette del Risco Sánchez, Erika Zambrano, Larissa Rodrigues, Nathália Quitério Daluia, Fernanda Garanhani Surita

**Affiliations:** ^1^ Department of Obstetrics and Gynecology, School of Medical Sciences Universidade Estadual de Campinas Campinas Brazil; ^2^ School of Nursing Universidade Estadual de Campinas Campinas Brazil; ^3^ School of Medical Sciences Universidade Estadual de Campinas Campinas Brazil

**Keywords:** gender‐based violence, health promotion, screening, women's health services

## Abstract

**Background:**

Violence against women remains a serious public health problem and a violation of human rights that affects women's health. Healthcare providers play a fundamental role in preventing and responding to violence against women and girls.

**Objectives:**

This study analyzes strategies for preventing gender‐based violence in healthcare services, focusing on identifying best practices for implementing evidence‐based interventions in these settings.

**Search Strategy:**

The review was conducted following the Joanna Briggs Institute's method for umbrella reviews and the Evidence Synthesis for Health Policy. A comprehensive search strategy was applied across eight databases.

**Selection Criteria:**

Secondary studies of interventions that address gender‐based violence in healthcare settings for women and adolescents of reproductive age were included in this review. Two reviewers independently screened the studies and assessed their quality.

**Data Collection and Analysis:**

Narrative synthesis was performed to summarize the evidence.

**Main Results:**

A total of 24 systematic reviews conducted in healthcare settings were analyzed, most of them from high‐income countries with diverse tools and methods. Screening strategies are implemented, particularly among at‐risk populations in healthcare settings most frequented by women, such as sexual and reproductive services. Initiatives that include educational elements, counseling, and advocacy interventions show promising results.

**Conclusion:**

Collaborative strategies in healthcare settings that guarantee the follow‐up of the survivors, the mitigation of gender‐based violence consequences, and its intergenerational transmission are necessary. Culturally sensitive strategies based on women‐centered, diversity, and equity approaches must guide the implementation of the interventions.

## 1 INTRODUCTION

### 1.1 Background

Violence against women (VAW) is considered by the World Health Organization (WHO) to be a serious public health problem and a violation of human rights, stemming from gender inequality.^1^ According to recent data from the WHO, 25% of women aged 15 to 49 years in the Americas have experienced sexual and/or physical violence at some point in their lives.^2^ Intimate partner violence (IPV) and non‐partner sexual violence are the most common forms of VAW worldwide.^3^

Globally, the eradication of any form of violence is a priority linked to the UN Sustainable Development Goals. Several of these goals and their targets promote commitments to prevent gender‐based violence (GBV), such as Goal 5, Achieve gender equality and empower all women and girls; Goal 3, Ensure healthy lives and promote well‐being for all at all ages; and Goal 10, Reduce inequality within and among countries.^2^

Continued exposure to various forms of violence has adverse effects on women's health, including depression, anxiety, post‐traumatic stress, insomnia, eating disorders, substance abuse, and suicidal behavior.^4^, ^5^ Other consequences are unintended pregnancies, miscarriage, gynecological problems, and sexually transmitted infections, with an increased risk of HIV infection.^6^, ^7^, ^8^

The health sector is recognized as a fundamental entry point for survivors of violence in high‐middle‐ and low‐income countries.^8^, ^9^ The increasing demand for health services among survivors of violence underscores the need for evidence‐based interventions in healthcare settings to address these issues.^8^

The WHO recommendations indicate that health services should provide safe environments for addressing GBV.^10^ However, this requires evidence to identify effective prevention strategies suitable for healthcare contexts.

The development of this review constitutes an opportunity to enhance efforts to identify women and adolescents at risk of violence within healthcare services, thereby facilitating their access to specialized resources in their community settings. We aimed to analyze strategies for preventing GBV in healthcare services, focusing on identifying best practices for implementing interventions in these settings.

## 2 AIMS OF THE REVIEW

The aims of this review are:

- to identify strategies for preventing GBV in healthcare services for women and adolescents.
- to discuss evidence‐based recommendations to develop violence GBV prevention strategies within healthcare services.

### 2.1 Review question(s)

The review questions are:

- What types of strategies for preventing GBV have been implemented in healthcare services for women and adolescents?
- What are the potential benefits and harms of these interventions for the target audience?

## 3 METHODS

This review was conducted by combining the Joanna Briggs Institute (JBI) method for umbrella reviews^11^ and the Evidence Synthesis for Health Policy.^12^, ^13^ These methods will allow us to examine the evidence produced through systematic reviews comprehensively and make recommendations for healthcare practice, research, and health policies.

This review is the second of three developed as part of the project approved under Proposal Call No. 21/2023 – Transdisciplinary Studies in Public Health, supported by the National Council for Scientific and Technological Development – CNPq and the Department of Science and Technology of Secretariat of Science, Technology, Innovation and Health Complex of Ministry of Health of Brazil. The methods were approved by these institutions through systematic meetings that contributed to validating the procedures guiding the development of this review. The protocol was registered retrospectively in the Open Science Framework (10.17605/OSF.IO/4NTUE).

### 3.1 Eligibility criteria

This review considered secondary studies that included interventions to address GBV in healthcare settings focused on women and adolescents of reproductive age (10–49 years old). The age group for adolescents was selected based on WHO criteria for adolescents aged 10 to 19 years.^14^ Studies that included perpetrator‐based interventions or those with couples were not considered.

Primary, secondary, and tertiary levels of prevention are considered according to a broad definition that includes diverse forms of interventions that allow the early identification within healthcare settings of women and girls who are exposed to violence (e.g., routine screening, home visitation), the initial response to disclosure or identification (e.g., first‐line support, referral to legal and other support services), and the ongoing response (e.g., advocacy, safety planning, motivational interviewing).^8^

Those interventions conducted exclusively in a humanitarian context were not included due to the characteristics of these scenarios that demand specific strategies to produce an appropriate response to GBV. Those interventions in which GBV against women and adolescents was not the main outcome, whether the outcomes of the interventions were not accessible, as well as those that only focused on psychological or therapeutic interventions not observing a reduction in GBV exposure, were excluded. Those that combined interventions mainly focused on other health conditions like HIV prevention or drug and alcohol prevention programs but did not detail the main outcomes related to GBV.

Regarding the context, we included evidence produced within healthcare settings and their levels of healthcare (primary, secondary, and tertiary). We included literature from high‐income countries and low/middle‐income countries (LMICs); however, we highlight the regions where the evidence was collected, observing the potential contribution to strengthen the healthcare response to GBV in LMICs.

Secondary studies, including systematic reviews with or without meta‐analysis, and qualitative and mixed methods reviews were included. Other types of publications such as conceptual or opinion articles, observational studies, unpublished studies, and those that described a preliminary study were not included.

### 3.2 Search strategy

The following key terms were combined to elaborate the strategy: (Women OR Female) AND (“Domestic Violence” OR “Violence Against Women” OR “Gender‐Based Violence” OR “Intimate Partner Violence”) AND (“Mass Screening” OR Interventions OR Intervention OR Prevention OR Preventing OR Prevent) AND (“Systematic Review” OR “Meta‐Analysis” OR “Network Meta‐Analysis) (Table S1). An initial limited search of PubMed was undertaken to conduct a preliminary analysis of the text words contained in the title and abstract of the papers and of the index terms used to describe them.

The search strategy was elaborated by a health science librarian and researchers experienced in the development of this type of study. Search terms were defined according to DeCS/MeSH (Descriptors in Health Sciences/Medical Subject Headings) and free terms to make the search queries exhaustive. The final search was conducted in October 2024. The electronic database included PubMed, Health Systems Evidence, *Biblioteca Virtual em Saúde*, Scopus, Web of Science, Embase, Cochrane Library, and Cinahl without a temporal restriction. The search strategy was adapted to each database included.

Only studies published in English, Portuguese, and Spanish were included because of the research team's language skills.

### 3.3 Study screening and selection

All the papers identified were imported to the Rayyan Qatar Computing Research Institute software (Rayyan QRI. https://www.rayyan.ai), and any duplicates were removed. The selection process was performed through a blind and independent assessment by two researchers according to the eligibility criteria. For the pre‐selection of the papers, the titles, keywords, and abstracts were read by two reviewers following training meetings regarding the review protocol.

The selection criteria were tested with a sample of 25 titles/abstracts. The pre‐selected texts were also read in full by two researchers (EZ and ORS), assessing their correspondence according to the importance of the publications and the inclusion criteria. Divergences in the selection process were solved by consensus and a third reviewer (FGS) was consulted, if necessary.

### 3.4 Assessment of methodological quality

We used the JBI Critical Appraisal Checklist for Systematic Reviews and Research Syntheses to assess the methodological quality of systematic reviews.^11^ This tool includes 11 questions regarding the review's methodology, search strategy, and bias. Two researchers independently reviewed all included studies. We did not exclude any studies based on the quality assessment; however, the study quality was considered in data analysis, and the research team discussed their potential impact on the results (Table S2).

### 3.5 Data extraction and synthesis

We use a modified version of the standardized model from the Guideline for Evidence Synthesis for Health Policy.^13^ The following information was systematically collected: title, type of intervention, author(s) and year of publication, authors' country, publication date, study aims, study design of the included studies, data analysis method, participants or target population, healthcare setting of the studies, type of violence addressed, main findings, number of studies included in the review, proportion of studies conducted in low‐ and middle‐income countries, proportion of studies focusing on the interventions of interest, last year of search, limitations, and recommendations.

The papers were exported to a database in Microsoft Excel. To ensure the consistency and reliability of the data extracted, one of the reviewers (ORS) performed the data extraction, and a second reviewer verified the process (EZ). The data extraction form was revised, discussed, and validated by the team.

Narrative synthesis was performed to identify, characterize, and summarize the evidence. The collected data were organized through a synthesis matrix. The team discussed best practices based on the characteristics of the prevention strategies and outcomes reported by the authors.

## 4 RESULTS

**FIGURE 1:**
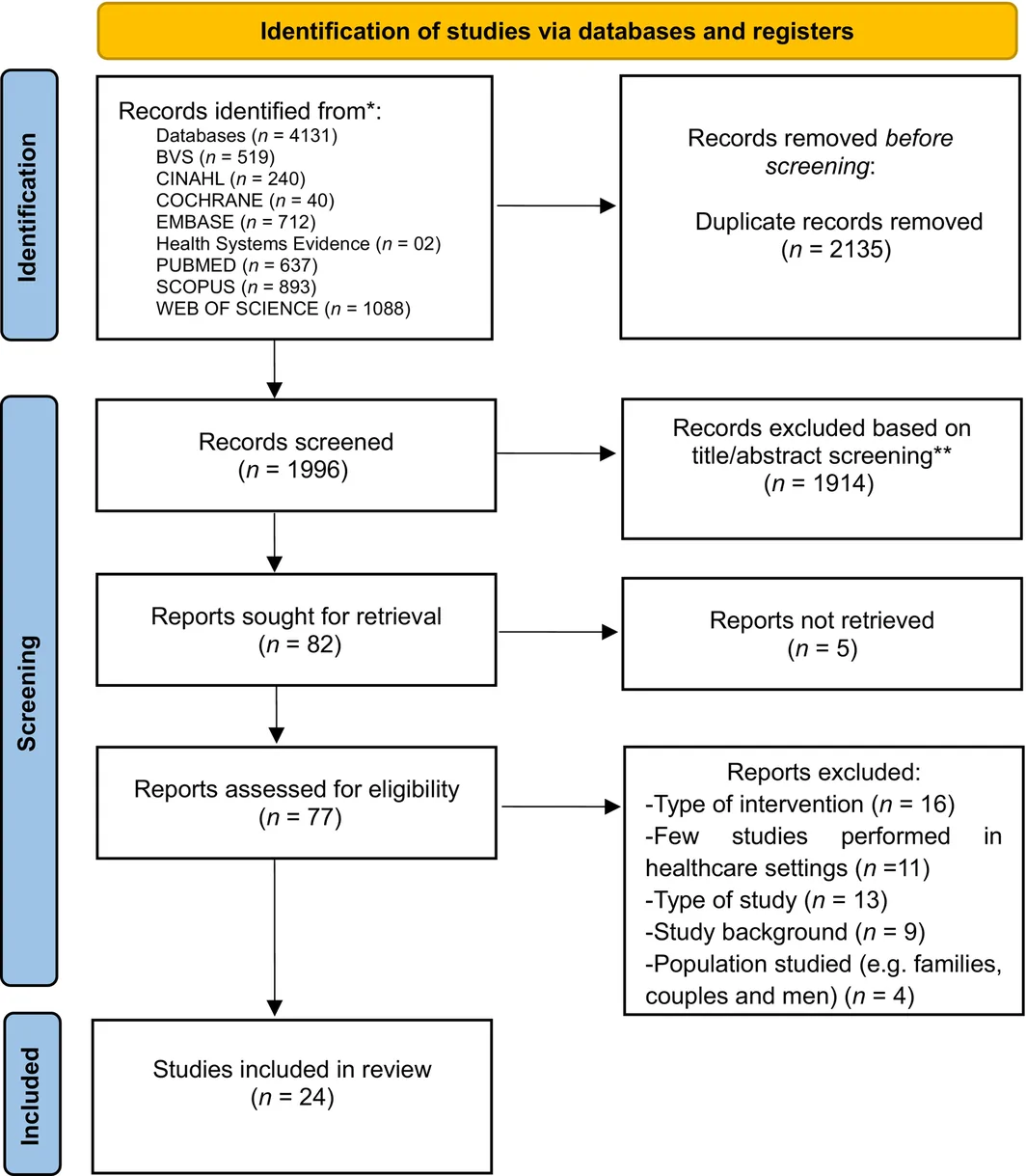
PRISMA flowchart.

In total, 4131 articles were retrieved, and 2135 duplicates were removed. A total of 1996 articles were screened, 77 were retrieved for full‐text reading, and 24 were kept for analysis. Figure 1 shows details of the selection process. The list of excluded studies and their reasons are presented in the Table S3.

### 4.1 Study characteristics

A detailed description of the included articles is presented in Table 1. The reviews were published between 2004 and 2024, and all of them were published in English. Seven reviews only include randomized clinical trials,^15^, ^16^, ^17^, ^18^, ^19^, ^20^, ^21^ and one includes only qualitative studies.^22^ Most of the reviews included primary studies of any design (*n* = 15). The data analysis was conducted through qualitative synthesis of the evidence (*n* = 18), meta‐analysis (*n* = 4), and a combination of quantitative and qualitative methods (*n* = 2) (Table 1).

**TABLE 1:** Characteristics of the studies included.

#	Author(s)/year/country	Type of intervention	Participants	Study design included	Data analysis method	Studies included	Proportion of studies focusing on the interventions of interest	Proportion of studies conducted in LMICs	Last year of search
1	Hussain et al., 2015 Canada/USA	Screening (ICT/ face‐to‐face and self‐administered tool)	Adult women (>18 years of age) attending healthcare setting	Randomized controlled trials	Meta‐analysis	6	6	0	2011
2	Adams et al., 2022 Australia	Home‐visiting	Home‐ visiting programs recipients	Qualitative studies	Thematic synthesis	26	26	0	2021
3	Prosman et al., 2015 The Netherlands	Home‐visiting	Abused women with children	RCT	Narrative synthesis	6	6	0	2014
4	Jahanfar et al., 2014 Canada	Brief individualized consultation, case management and referral to social care workers, and multiple therapy sessions	Pregnant women	RCTs including cluster‐randomized trials, and quasi‐randomized controlled trials	Meta‐analysis	10	8	1 (Peru)	2014
5	Sapkota et al., 2019 Australia/Nepal	Supportive counseling and mentoring	Pregnant women 18 years and above living in LMICs	Randomized trials and quasi‐experimental trials	Narrative synthesis	5	5	5 (Peru, South Africa, India, Kenya)	2018
6	Daley et al., 2020 UK	Screening, referral and supportive counseling	Women during pregnancy, childbirth or up to 12 months postnatal in LMIC	Primary studies of any design	Narrative synthesis	6	6	6 (Kenya, Peru, Nigeria, South Africa and India)	2019
7	Lewis et al., 2022 UK/Sri Lanka/Brazil/Nepal/Palestine/Switzerland	Complex interventions (first‐ line support, referrals, psychosocial counseling, coping skills training, and psychoeducation)	Recipients of healthcare services—women of reproductive age (15–49 years old) or providers of healthcare services in LMIC	Primary intervention studies of any design	Concurrent narrative quantitative and thematic qualitative syntheses, integration through line of argument and mapping onto a logic model	26	26	26 (sub‐Saharan Africa, Middle East, South Asia, and South America)	2019
8	Linde et al., 2020 Denmark	eHealth interventions	Women recipients of healthcare services and other specialized services	RCT	Meta‐analysis	14	8	0	2019
9	Van Parys et al. 2014 Belgium	Home visitation and counseling	Pregnant women of any age and/or women who had given birth in the past year	RCT	Qualitative synthesis	9	9	1 (Peru)	2013
10	Sabloak et al., 2024 USA	Provider‐facilitated discussions, a safety card and referral	Individuals who were seeking abortion services	Studies of any design	Narrative synthesis	9	9	0	2021
11	Bair‐Merritt et al., 2014 USA	Primary care‐based interventions Empowerment, provider‐facilitated discussion, and referral	Women recipients of primary healthcare services	Primary studies of any design	Narrative synthesis	17	17	2 (Peru and South Africa)	2012
12	O'Reilly et al., 2010 USA	Screening, resource card, counseling and mentoring intervention	Pregnant women attending antenatal care	Primary studies (historical control design, RCT, case–control)	Narrative synthesis	9	9	0	2009
13	Teichman et al., 2023 USA/ Saudi Arabia.	Screening, indirect questions, computer‐based health‐risk assessment	Emergency services and trauma center patients	Primary studies (prospective cohort studies, case–control)	Narrative synthesis	7	7	0	2022
14	Miller et al., 2021 USA	Screening	Healthcare services attendees	Primary studies of any design	Descriptive analysis of quantitative data and qualitative analysis based on implementation‐oriented frameworks	59	59	5 (India, Lebanon, Turkey, Iran, and Guinea)	2020
15	Trabold, 2007 USA	Screening, safety interventions and advocacy	Healthcare services attendees	Primary studies (experimental, nonexperimental, quasi experimental)	Qualitative synthesis	10	10	0	N/A
16	Kataoka, et al., 2004 Japan	Screening, counseling and advocacy	Pregnant women	Primary studies (RCT, non‐equivalent control group study and two time‐series studies) and systematic reviews	Qualitative synthesis	12	10	0	2003
17	Nelson et al., 2012 USA	Screening, home visitation, nursing case management, counseling, wallet‐sized referral card and mentor support	Women presenting for health care without problems directly related to abuse	Trials of the effectiveness of screening and interventions, diagnostic accuracy studies of screening instruments, and studies of any design about adverse effects	Qualitative synthesis	Screening effectiveness *n =* 1 Screening techniques *n =* 15/ Interventions *n =* 6 Adverse effects *n =* 14	22	1 (Brazil)	2012
18	Basheer et al., 2022 Great Britain	Advocacy	Survivors of domestic abuse in emergency departments	Primary studies (RCT and observational studies)	Qualitative synthesis	5	5	0	2022
19	Howell et al., 2017 USA	Screening, advocacy, counseling, multi‐session care, and nurse case management	Pregnant women	Primary intervention studies of any design	Qualitative synthesis	17	17	1 (Peru)	2016
20	Tompsett et al., 2024 UK	Technology‐based screening tools	People experiencing domestic abuse and/or people experiencing modern slavery	Primary studies and practice guide	Narrative synthesis using the convergent integrated approach	24	20	0	2023
21	Anderson et al., 2021 USA	mHealth Interventions	Adults or adolescents in adult romantic relationships	Empirical studies (RCT, quantitative nonrandomized, quantitative descriptive, qualitative, and mixed‐methods)	Qualitative synthesis	31	24	1 (Cambodia)	2019
22	Ahmad & Mahmood, 2010 Canada	Computer‐assisted interactive screening	Adult populations	Empirical studies (descriptive, randomized trial, and qualitative)	Qualitative synthesis	4	4	0	2010
23	O'Doherty et al., 2015 Australia	Screening (face‐to‐face, written, computerized tool)	Women over 16 years of age attending any healthcare setting	Randomized or quasi‐randomized controlled trials	Meta‐analysis and narrative description	13	13	0	2015
24	El Morr & Layal, 2020 Canada	ICT interventions	Women who experienced intimate partner violence or domestic violence	Primary studies of any design	Qualitative synthesis	25	25	0	2020

Abbreviations: ICT, information and communication technologies; LMIC, low/middle‐income countries; N/A, not available; RCT, randomized controlled trial.

Many of the reviews were produced by authors from high‐income countries. Out of the 24 reviews, 21 collected data from studies performed in high‐income countries, and 10 included almost one primary study from LMICs (Table 1).

**FIGURE 2:**
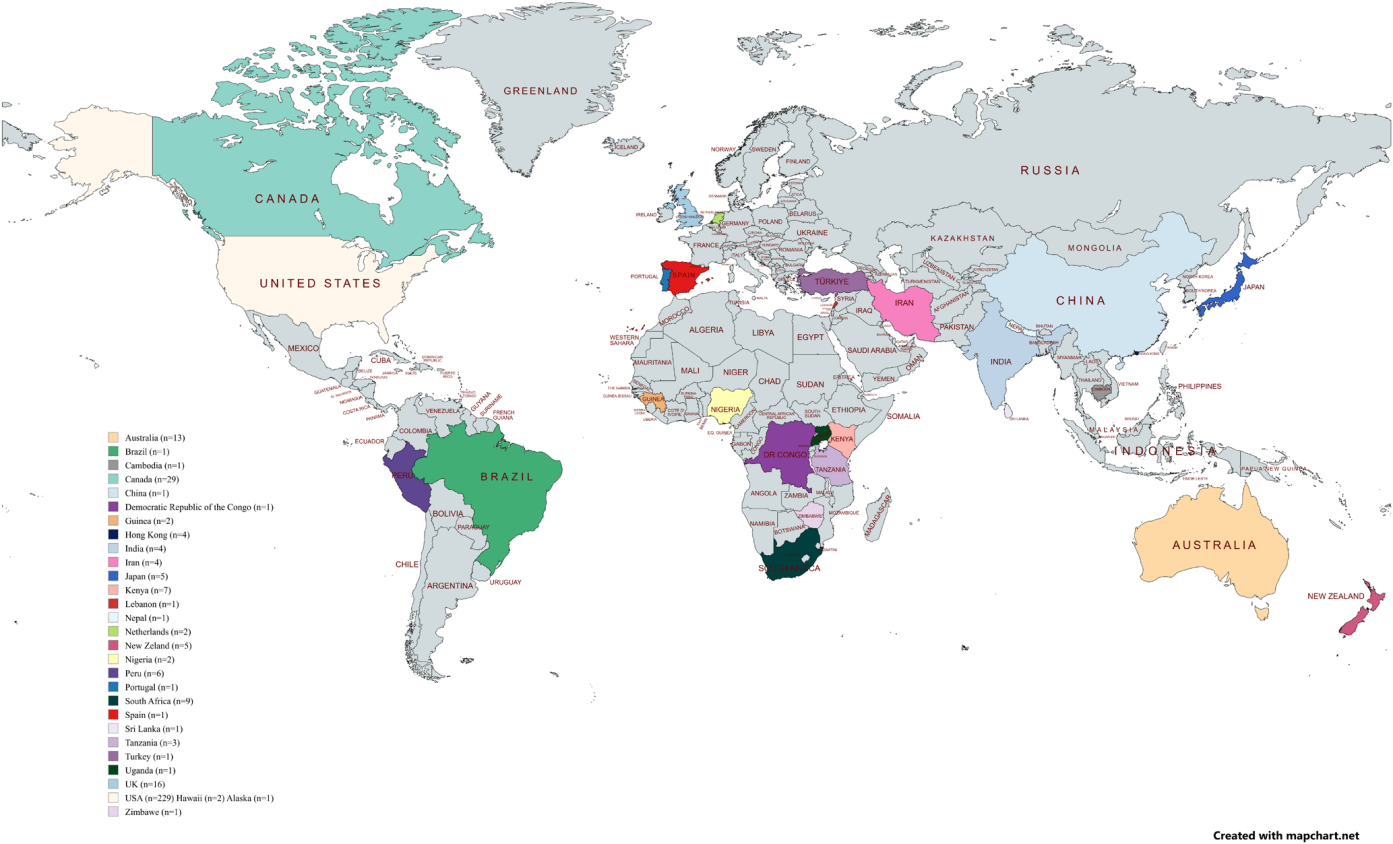
Countries of primary studies with interventions of interest analyzed in 24 systematic reviews.

Overall, 359 primary studies about interventions of interest were included in the systematic reviews, most of which were conducted in US healthcare settings. Three studies were conducted in multiple settings (Jordan, Kenya, Ethiopia, and Congo *n* = 1; South America *n* = 1; USA, Canada, and the UK *n* = 1) (Figure 2).

Regarding the population, some studies included only pregnant and/or postpartum women (*n =* 7), women who experienced or were at risk of IPV or domestic violence (DV) exposure (*n =* 5), attendees of healthcare settings (*n =* 11), and home‐visiting programs (*n =* 1).

Additionally, 14 articles studied IPV,^15^, ^17^, ^18^, ^19^, ^23^, ^24^, ^25^, ^26^, ^27^, ^28^, ^29^, ^30^, ^31^, ^32^ five papers DV,^16^, ^33^, ^34^, ^35^, ^36^ and one article used both terms.^23^ Another article studied DV and forms of modern slavery.^30^ Other terms used are family violence,^22^ perinatal violence,^20^ as well as VAW as a broader term, including any type of violence against a woman, including transgender women^3^ (Table 2).

**TABLE 2:** Main findings of the systematic reviews.

#	Author (s) /year/country	Study aims	Type of violence	Healthcare settings	Main findings
1	Hussain et al., 2015 Canada/USA	To assess the rate of IPV disclosure in adult women (>18 years of age) with the use of three different screening tool administration methods: computer‐assisted self‐administered screen, self‐administered written screen, and face‐to‐face interview screen	IPV	General/family practice, ED, women's health clinic, pediatric care, ANC/postnatal services	Computer‐assisted self‐administered screens results in higher rates of IPV disclosure in comparison to both face‐to‐face interview and self‐administered written screens
2	Adams et al., 2022 Australia	To systematically review and synthesize qualitative research exploring home‐ visiting nurses' roles and identify the challenges for nurses working with women experiencing family violence	FV	Family health and support programs	Two themes emerged (1) relationship building—with the client, with services and with colleagues/self; and (2) family violence practice—ask/screen, validate/name, assess risk/safety plan and safeguard children
3	Prosman et al., 2015 The Netherlands	To assess the effectiveness of home visiting in reducing IPV experienced by mothers	IPV	ANC, gynecological care, PHC and pediatric care	Three of the six home visiting programs, focused on reducing IPV for mothers, showed a statistically significant reduction of IPV in the short‐term. However, it is not known whether these results are effective in the long term
4	Jahanfar et al., 2014 Canada	To examine the effectiveness and safety of interventions in preventing or reducing domestic violence against pregnant women	Perinatal violence	Healthcare services/ANC and community‐based services	There is insufficient evidence to assess the effectiveness of interventions for domestic violence on pregnancy outcomes. The authors did not find any evidence that interventions had a negative, harmful effect
5	Sapkota et al., 2019 Australia/Nepal	To obtain a complete representation of interventions available in LMICs for reducing and/or controlling DV among pregnant women and assess their effectiveness	DV	Healthcare services and community‐based services	Supportive counseling interventions demonstrated a reduction in DV and an improvement in use of safety behaviors. A context‐specific intervention that address the interplay of multiple factors are more likely to have positive effects on reducing DV and managing its health consequences. The development of a theory of change is recommended
6	Daley et al., 2020 UK	To review systematically the available evidence describing types of interventions for, and/or effectiveness of such interventions for women living in LMIC, who report domestic violence during and/or after pregnancy	DV	ANC, PHC, local community, healthcare facility for HIV post‐test counseling	Screening, referral and supportive counseling is likely to benefit women living in LMIC who experience DV. High‐quality studies to provide evidence for the effectiveness of these interventions are lacking
7	Lewis et al., 2022 UK/Sri Lanka/Brazil/Nepal/Palestine/Switzerland	To synthesize evidence on the effectiveness, cost‐ effectiveness and barriers to responding to violence against women (VAW) in sexual and reproductive health (SRH) services in low/middle‐ income countries (LMICs)	VAW/IPV/DV/FV	SRH services in LMICs	The interventions were categorized into: (1) Response to VAW during routine SRH consultation, (2) Response to VAW during routine SRH consultation plus community, and (3) Response to VAW in addition to routine SRH consultation. Accepting attitudes towards VAW, fear of consequences, and limited readiness of the society, health systems, and individuals influence the outcomes of the interventions
8	Linde et al., 2020 Denmark	To examine the effect of eHealth interventions compared with standard care on reducing IPV, depression, and posttraumatic stress disorder (PTSD) among women exposed to IPV	IPV	Health clinics, mental health services, pediatric emergency services, FV units	There is no evidence that eHealth interventions reduced physical, sexual, or psychological violence, depression, or PTSD compared with no eHealth intervention
9	Van Parys et al. 2014 Belgium	To provide a clear overview of the existing evidence on effectiveness of interventions for IPV around the time of pregnancy	IPV	ANC services	The studies did not produce strong evidence that certain interventions are effective. Home visitation programs and some multifaceted counseling interventions did produce promising results. There is no reported evidence of harmful effects from the interventions
10	Sabloak et al., 2024 USA	To perform a systematic review of screening tools and interventions focused on reducing adverse health outcomes associated with intimate partner violence (IPV) at abortion‐related visits	IPV	Family planning services	Appropriate interventions screening for IPV improve the patient–provider relationship and connects patients with integral IPV‐related resources in family planning healthcare settings
11	Bair‐Merritt et al., 2014 USA	To summarize evidence describing primary care–based interventions for patients experiencing IPV	IPV	PHC	Most studies showed patient‐level benefits following primary care IPV interventions, with IPV/community referrals being the most commonly positively affected outcome
12	O'Reilly et al., 2010 USA	To appraise the effectiveness of DV screening and interventions for women identified for DV through screening in pregnancy	DV	ANC services and public health clinics	Among the screening studies, DV identification rates were significantly higher compared to those using nonstandardized screens or no screening at all. Routine screening throughout pregnancy further increased identification rates
13	Teichman et al., 2023 USA/ Saudi Arabia.	To review research on current practices for IPV prevention in emergency departments and trauma centers in the USA and provide evidenced‐based recommendations	IPV	ED, Trauma center, Emergency room	Screening instruments are diverse but is unclear if one tool is more accurate than others. Universal screening and the use of screening protocols improved IPV victim identification. No studies reported immediate adverse effects with screening
14	Miller et al., 2021 USA	To investigate the utility of IPV screening administered by frontline clinical personnel	IPV	PHC, ED, maternal and child health programs	The median reach of the IPV screening programs was high (80%), but ED settings were found to have a much lower reach (47%). The lack of available referral services affected the implementation of IPV screening
15	Trabold, 2007 USA	To present literature regarding intervention research on Intimate Partner Violence (IPV) screening	IPV	OB/ GYN clinic, PHC, ED, pediatric primary care	Screening is effective in identifying IPV; however, a causal link between screening practices, increased safety practices, and decreased violence cannot be established
16	Kataoka et al., 2004 Japan	To review published studies focusing on the screening of DV against women, in particular, the instruments, the screening methods and the interventions used to help abused women after screening	DV	Prenatal clinics, public clinics and ED	Contrasting differences in the reported effectiveness of the screening methods (face‐to face interview or written questionnaire) were observed. Counseling sessions after screening and the advocacy program for postsheltered women were effective in reducing DV
17	Nelson et al., 2012 USA	To review new evidence on the effectiveness of screening and interventions for women in health care settings in reducing IPV and related health outcomes, the diagnostic accuracy of screening instruments, and adverse effects of screening and interventions	IPV	PHC, ED, family‐planning clinics and student health centers	Screening instruments accurately identify women experiencing IPV. Screening women for IPV can provide benefits that vary by population, while potential adverse effects have minimal effect on most women
18	Basheer et al., 2022 Great Britain	To evaluate the evidence for before/after studies and randomized control trials looking at the effectiveness of such advocacy work for victims presenting in the ED to determine if they reduce abuse related co‐morbidities, re‐admission rates, use of community/police resources, and among other outcomes	DV	ED	Despite weak evidence, studies suggest that advocacy interventions tend to be helpful, unlikely to be harmful, but are of uncertain cost‐effectiveness
19	Howell et al., 2017 USA	To present representative literature on the effects of IPV in pregnancy, conduct a systematic review of existing interventions for IPV‐exposed pregnant women and provide recommendations for future translational research in this area	IPV	Healthcare services, ANC	Interventions for IPV‐exposed pregnant women are scarce and largely limited to crisis intervention approaches. Available interventions seeking to address broader or intergenerational effects of violence are limited in scope, and effectiveness data are preliminary
20	Tomsett et al., 2024 UK	To explore the technology‐based tools available for supporting the identification of victims of domestic abuse and modern slavery in remote services and consider the benefits and challenges posed by the existing tools	DV and modern slavery	Remote healthcare services (ED, PHC, Women's health clinics)	Remote technology can serve as an acceptable and practical tool for screening abuse victims by both service users and healthcare professionals. When used to complement face‐to‐face approaches, it may lead to higher overall screening rates and victim identification
21	Anderson et al., 2021 USA	To identify the full spectrum of mHealth interventions that are designed for IPV victim use and provide insight into which populations are being served by mHealth interventions to prevent IPV	IPV	Outpatient medical services, psychology/therapy, community organization	Computer‐based screening, with or without integrated education, was the most common mHealth approach, followed by safety decision aids. Studies that assessed feasibility and acceptability reported high levels of both. There was limited evidence on whether mHealth interventions addressed population needs more effectively than conventional approaches. mHealth tools for IPV prevention are particularly well‐accepted in healthcare settings, on mobile phone platforms, and for connecting victims to healthcare services. These strategies can tailor interventions to individual victim needs without requiring extensive provider resources
22	Ahmad & Mahmood, 2010 Canada	To critically review current scientific knowledge on the use of enhanced Web 2.0 interactive computer‐assisted screening for IPVC in clinical settings	IPVC	ED and family medicine settings	Computer‐assisted screening in similar settings can improve the detection and disclosure of IPVC. However, a coordinated multiservice response is essential for a comprehensive approach. Time‐constrained frontline clinicians require a more supportive environment for managing detected IPVC cases, with an even greater need for system‐level support in emergency settings
23	O'Doherty et al., 2015 Australia	To assess the effectiveness of screening for IPV conducted within healthcare settings on identification, referral, re‐exposure to violence, and health outcomes for women, and to determine if screening causes any harm	IPV	ANC, women's health, maternity services, ED and PHC	Pregnant women in antenatal settings may be more likely to disclose IPV when screened; however, robust evidence is still needed. While screening increases the identification of IPV in healthcare settings, there is insufficient evidence to support universal screening. A more effective approach may be to train healthcare professionals to identify and inquire about abuse in women showing signs of violence or those in high‐risk groups. Additionally, providing a supportive response, relevant information, and safety planning can enhance care for those affected
24	El Morr & Layal, 2020 Canada	To examine whether ICT could become acceptable for effective IPV interventions, we reviewed the literature on the use of ICT based interventions to address IPV issues	IPV/DV	Healthcare services (ED, pediatric emergency departments, ANC, community health centers, trauma treatment centers, and family practices)	Research addressing safety, equity, and the unintended consequences of the use of ICT in IPV programming is limited. Technology is costly in terms of hardware, software, and data plan costs, being challenging, particularly for LMICs. Studies found that IPV disclosure through ICT was generally more effective than face‐to‐face disclosure. It was perceived as less judgmental and more anonymous, which encouraged greater disclosure

Abbreviations: ANC, antenatal care; DV, domestic violence; ED, emergency department; FV, family violence; IPV, intimate partner violence; IPVC, intimate partner violence and control; OB/GYN, obstetric and gynecology; PHC, primary health care; SRH, sexual and reproductive health; VAW, violence against women.

For the presentation of results, thematic categories were constructed to summarize the prevention strategies explored. The findings were focused on three major approaches regarding the level of prevention: (1) Primary Interventions: Strategies for identification of women and girls who are exposed to GBV; (2) Secondary interventions: Initial response to disclosure or identification of GBV; (3) Tertiary interventions: Ongoing response to GBV. The potential benefits and harms of the interventions were also described.

### 4.2 Primary interventions: Strategies for early identification of women and girls who are exposed to gender‐based violence

Gender‐based violence (GBV) screening in healthcare services is used to prevent VAW using diverse measurements. A total of 15 studies examined the effects of screening (e.g., reducing violence) and/or compared different screening methods.^19^, ^21^, ^23^, ^24^, ^26^, ^27^, ^28^, ^29^, ^30^, ^31^, ^32^, ^33^, ^34^, ^35^, ^37^ Overall screening is recognized as a practice that increases the identification of DV/IPV victims.^21^, ^26^, ^28^, ^34^ The administration methods varied among face‐to‐face, self‐administered, and remote interventions.

Healthcare settings such as emergency departments (ED) emerged as a possible scenario to implement screening strategies conducted by nurses, physicians, social workers, and other healthcare practitioners.^19^, ^21^, ^23^, ^26^, ^27^, ^28^, ^30^, ^31^, ^32^, ^35^ Clinics specialized in women's health including reproductive health services, antenatal and postnatal care services, obstetrics and gynecology clinics, maternal and childcare programs as well as pediatric services were also mentioned.^19^, ^21^, ^24^, ^27^, ^28^, ^29^, ^30^, ^32^, ^33^, ^34^, ^35^

Additionally, public health clinics, family medicine settings, healthcare facilities for HIV post‐test counseling, primary healthcare, and community‐based services^19^, ^21^, ^23^, ^27^, ^28^, ^29^, ^30^, ^31^, ^33^, ^35^, ^37^ were scenarios where screening was implemented.

Among the type of screening methods used in the studies, strategies focused on^21^:

- Universal screening: A standardized question is applied consistently to all asymptomatic women^21^, ^26^, ^28^
- Selective screening: Targets high‐risk groups such as pregnant women or those seeking pregnancy termination^21^, ^23^, ^28^, ^29^, ^31^, ^33^, ^34^, ^35^
- Routine enquiry: All women are asked about their experiences periodically^21^, ^31^, ^33^, ^35^
- Case‐finding: The screening is conducted only if specific symptoms or signs are present (e.g., women attendee to the ED with vaginal bleeding).^21^, ^28^

Among the instruments used to screen, the following are observed:

- Use of standardized and validated questionnaires, which might also be culturally adapted in certain cases (e.g., Woman Abuse Screening Tool (WAST), Partner Violence Screen (PVS), Hurt, Insult, Threaten, and Scream (HITS), Danger Assessment Scale, Abuse Assessment Screen, Conflicts Tactics Scales (CTS), Abuse Assessment Screen)^19^, ^21^, ^23^, ^26^, ^27^, ^29^, ^33^, ^34^, ^35^, ^37^
- Health risk assessment evaluation^26^, ^33^
- Remote strategies (e.g., telephone surveys, computerized tools)^19^, ^21^, ^23^, ^26^, ^30^, ^31^, ^37^
- Use of direct and indirect questions and face‐to‐face conversations^21^, ^23^, ^26^, ^28^, ^30^, ^35^
- Custom IPV screening and implementation of IPV screening protocols.^26^, ^27^

One review of screening in ED and trauma center patients observed a variation among the screening tools, but it was unclear if one tool was more accurate than the other; however, direct questioning tends to capture the greatest number of victims of IPV.^26^

Another systematic review and meta‐analysis to assess the effectiveness of screening for IPV concluded that there is insufficient evidence to justify universal screening, and other practices might be more effective, such as enquiring about abuse signs in women or those in high‐risk groups, offering supportive responses, appropriate information, and support for plan safety.^21^

Another strategy for early identification of violence exposure is home‐visiting programs.^15^, ^18^, ^20^, ^22^, ^23^, ^27^, ^29^ Nurses, social workers, paraprofessionals, and mentor mothers mainly conduct these programs.

Despite these strategies being focused on early identification of violence, their components are not limited to the increase in the detection of GBV exposure among women and their children. These programs involved active listening and screening, contributing to naming the abuse experiences and validating women's experiences, assessing the risk, and helping to elaborate a safety plan and safeguard children.^22^ The potential for providing initial support, empowering, referring, and follow‐up is observed across these programs. Some of them start as part of antenatal care (ANC) and continue during postnatal care, reaching vulnerable groups including prevention of children's exposure to these forms of abuse.^15^, ^22^

Woman‐centered care, trauma‐informed practice, maternal and child interaction, and the social determinants of health are essential for the implementation of these programs.^22^ As well as professional skills like open conversational style, using open‐ended questions, non‐judgmental attitude, and observational skills allows the implementation of these programs.^22^

### 4.3 Secondary prevention: Initial response to disclosure or identification of gender‐based violence

Most of the review studies included a diverse range of interventions adopted for primary studies to provide an appropriate response to GBV survivors. The interventions aimed to offer an initial response to address GBV were:

- Empowerment interventions aimed to strengthen women's readiness to respond to GBV, abuse assessment, wallet‐size referral cards, psychoeducation, and psychosocial and supportive counseling^3^, ^16^, ^18^, ^20^, ^23^, ^24^, ^25^, ^29^, ^32^, ^33^, ^34^, ^35^
- Educative initiatives for women and community engagement actions to develop communicative and social skills (e.g., sexual negotiation, conflict resolution, gender norms), coping skills, knowledge about HIV prevention and gender‐relevant issues, discussion of the cycle of violence and safety^3^, ^16^, ^20^, ^23^, ^25^, ^29^;
- Providers' education focused on sensitive training for healthcare professionals to address GBV, improving their competencies in screening, supporting referrals, raising awareness, and promoting prevention^3^, ^16^, ^24^;
- Providing appropriate information and facilitating survivors' access to community‐based resources, legal services, and other support services.^3^, ^16^, ^20^, ^24^, ^25^, ^28^, ^33^

Overall, these secondary prevention interventions are brief and delivered by health clinic staff, nurses, trained social workers, counselors, community workers, IPV advocates, and other nonphysicians.^16^, ^25^

According to Bair‐Merritt et al.^25^ interventions often share key characteristics, including their focus on enhancing self‐efficacy and empowerment, improving access to IPV‐related resources, and utilizing brief intervention models delivered by nonphysician professionals based on empathetic listening.

### 4.4 Tertiary prevention: Ongoing response to gender‐based violence

Among the interventions focused on strategies to mitigate the adverse outcomes of GBV exposure and reducing re‐victimization, advocacy was one of the interventions reported by the primary studies.^28^, ^29^, ^35^, ^36^ The use of motivational interviewing and additional on‐site resources, such as a victim advocate or social worker were included in one review conducted in abortion‐related services.^24^ The motivational interviews targeted women who had experienced IPV, aiming to identify progressive goals and measures that enhance self‐efficacy and feelings of control.^24^

Safety interventions were examined in some reviews contributing to making a safety plan and accessing resources.^20^, ^23^, ^28^, ^35^ This initiative included the identification of actions or behaviors that help achieve or maintain decreased harm by a partner. This includes the use of community resources (e.g., shelters, counselors, and advocates) and involvement in safety planning (e.g., removing weapons from the home, preparing an emergency bag with clothing, securing a driver's license and bank account, and reporting incidents to the police).^28^ Advocacy is observed in these studies as provided by trained individuals addressing various needs like legal assistance, housing, and employment.^28^, ^35^

### 4.5 Potential benefits and harms of the interventions

Overall, there is evidence regarding the benefits of GBV interventions, but few studies refer to any concern with potential harm.

Screening in the ED and trauma center showed significant success in identifying IPV victims, and no immediate adverse effects related to screening were reported.^26^ Screening increases the rates of disclosure, creating more opportunities to offer resources, and has been associated with reduced violence, decreased isolation, and improved safety practices; however, authors are careful to affirm a causal link among these outcomes.^20^ The benefits and harmful effects of screening vary across populations; however, adverse effects generally have minimal impacts on women (e.g., discomfort, lack of privacy, emotional distress, and worries about further abuse).^32^

Despite few home visiting programs focused on IPV reduction, they show promising results in reducing IPV.^18^ However, it is not known whether these results are effective in the long term.^15^ Home visiting allows the provision of assistance to those vulnerable women with children who were hard to reach through regular services.^15^

Supportive counseling shows a reduction in the frequency and severity of DV, particularly physical and sexual abuse, and despite screening and referral showing behavior changes, those changes were not statistically significant.^33^

Supportive counseling and advocacy tend to show a reduction in DV and an improvement in the use of safety behaviors.^16^, ^28^, ^32^, ^35^, ^36^ Similarly, some evidence shows that counseling tends to improve quality of life, family and social support, ensure greater access to community resources, increase the use of referral services, and reduce maternal depression.^33^ A review regarding advocacy in ED shows their potential benefits to connecting “hidden” victims with essential resources, such as shelters and counseling services.^36^

However, contrasting findings are observed among reviews. For instance, out of six primary studies evaluating supportive counseling interventions, only two reported a statistically significant effect of the intervention on IPV.^18^ Similarly, when comparing the methods, another review concludes that the evidence was still mixed regarding whether the counseling intervention was more effective than the use of resource cards.^34^

In addition, interventions that focused on IPV providers' training showed improved outcomes by enhancing patient self‐efficacy and using non‐stigmatizing language, improving the patient–provider relationship while also connecting patients with essential resources.^24^

Among the interventions, in LMICs, intensive psychosocial support in addition to routine sexual and reproductive health consultations showed the strongest evidence for reducing the recurrence of violence and improving health outcomes due to enhanced safety behavior.^3^

## 5 DISCUSSION

**FIGURE 3:**
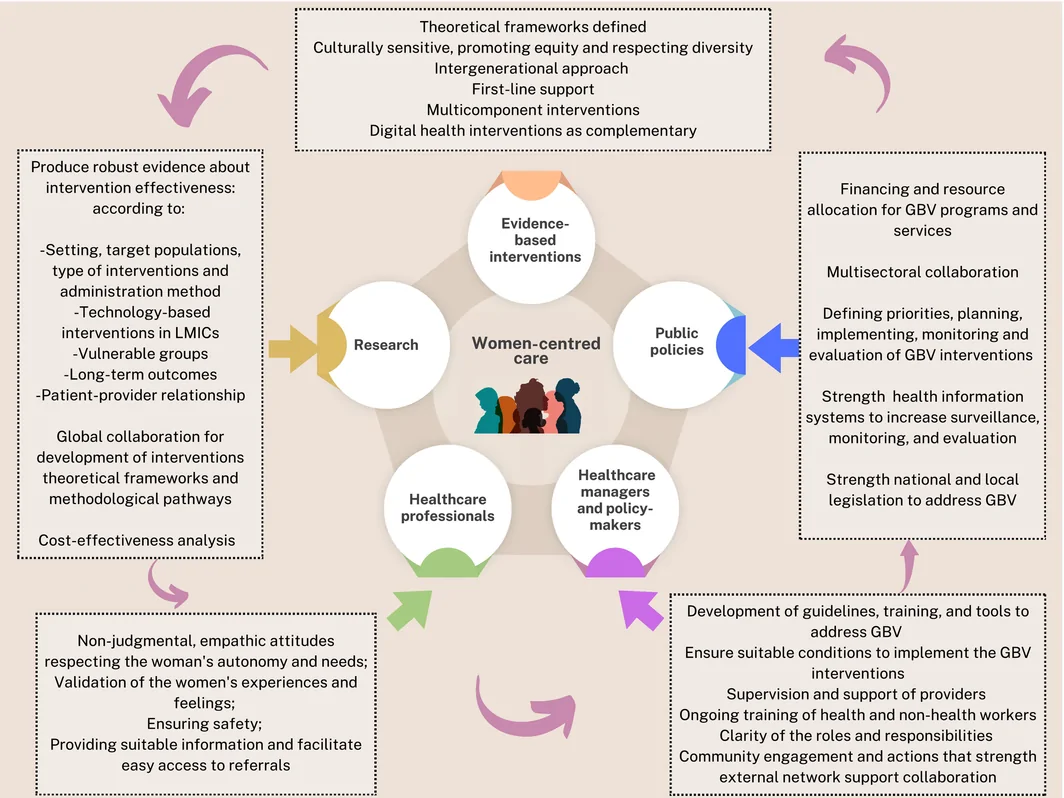
Recommendations for healthcare professionals and managers' practices, research, and public policies about gender‐based violence (GBV) interventions in healthcare settings.

Gender‐based violence interventions show a spectrum of tools and methods. Healthcare settings provide suitable opportunities for preventing GBV and addressing its effects. Several recommendations have been developed to enhance the practices of healthcare professionals, encourage research on this topic, and improve healthcare policies (Figure 3) (Table S4).

The studies focused on pregnant and/or postpartum women attending ANC services and women with vulnerability to past and/or current experience of violence from healthcare services such as family planning and abortion services, ED, PHC, and community healthcare services.

However, few primary studies have included pediatric care services or perinatal programs as settings for GBV interventions (*n =* 9 systematic reviews). Interventions based on a systemic and intergenerational approach are recommended as a comprehensive and feasible model for reaching pregnant women and their children.^29^

These highlight that those settings that are the entry point to the treatment of injuries (e.g., ED), reproductive healthcare services, and facilities within women's communities are suitable environments to incorporate protocols to address this issue due to the potential benefits of early identification, awareness of GBV, and first‐line support.

However, the implementation of changes in these environments is not a simple task. Healthcare providers must deal with issues like multiple roles performed, staff turnover, workload pressures, conflicting priorities, restricted time, and limited referral options among other context‐specific factors.^16^, ^22^, ^27^, ^38^ Future studies to explore whether the rate of IPV disclosure varies depending on the clinical setting and the method used to administer screening tools are recommended.^19^

The studies focus on interventions for GBV during women's life stages, particularly in pregnancy and the postpartum period.^25^, ^29^, ^33^, ^34^, ^35^ Evidence suggests that routine screening should be an encouraged practice for women of childbearing age.^25^

Professional organizations like The American College of Obstetricians and Gynecologists recommend routine inquiry during prenatal care at least once per trimester and during postpartum check‐ups.^39^ The Women's Preventive Services Initiative recommends screening adolescent and adult women for intimate partner and domestic violence, at least annually, and, when needed, providing or referring to intervention services.^39^ Moreover, these interventions must guarantee some conditions, such as protocol/standard operating procedures, a system for referral in place, training, and privacy and confidentiality.^10^

Systematic screening during the prenatal and postpartum periods, accompanied by appropriate interventions, is an effective strategy to prevent re‐exposure to violence. Evidence indicates that such screening causes no apparent harm and may empower women, strengthening their trust and relationship with healthcare providers.^40^

However, despite recommendations from the American College of Obstetricians and Gynecologists to implement systematic screening, uptake remains low in these settings. Barriers include lack of provider training, discomfort or uncertainty among professionals, the predominance of a biomedical model of care, and broader structural constraints such as limited time, lack of privacy, and insufficient support.^41^ Prior reviews have shown promising strategies for preventing GBV in challenging settings like LMICs.^3^, ^16^, ^33^ Out of 24 systematic reviews, only three explored LMIC contexts, and among those secondary studies that include these countries, primary studies from LMICs are scarce.

Additionally, the evidence is still conflicting regarding which screening tools and interventions show better outcomes.^19^, ^20^, ^26^ The definition of a “gold standard” is unclear, and most of researchers are cautious regarding recommendations, in most cases due to the low quality of the evidence and study heterogeneity.^3^, ^17^, ^18^, ^19^, ^20^, ^21^, ^26^

The findings suggest that the standardization of interventions will be considered with caution due to the heterogeneity observed in the findings among healthcare settings and regions. Flexible, culturally sensitive models that respect diversity and promote equity are essential for the implementation and monitoring of evidence‐based interventions.

Overall, contextual factors and system‐level measures potentially influence the success of the interventions. Screening initiatives that incorporate educational components for healthcare providers show promising results.^28^ Moreover, women's and professionals' acceptability and preferences regarding the tools and administration methods must be considered as essential components of the initiatives.

Another critical point is the length of the interventions. Most of the studies analyzed included brief interventions, except some home visiting programs that tend to be across antenatal and postnatal care. Brief interventions have some benefits associated with time management, but this model commonly does not comprehensively address survivors' needs.^29^

Further, some evidence reveals the potential benefits of the use of comprehensive and multifaceted interventions (e.g., when GBV is addressed and linked with other topics like HIV prevention and substance abuse programs) due to cost reductions and acceptability from users.^16^

Observing the evidence produced about the most effective strategies to reduce re‐victimization, the evidence reinforces that violence does not end when it is detected.^10^ Therefore, while screening strategies are important to increase surveillance after screening, subsequent interventions are required for any participant at risk.^15^, ^33^

Evidence indicates that cisgender women and adolescents aged 15 to 49 years, racialized women, women with disabilities, immigrants, pregnant women, individuals, and those living with stigmatizing health conditions (e.g., cancer, mental health conditions, and HIV), as well as people with gender or sexual identities that diverge from heteronormative norms, might experience various forms of GBV throughout their lives.^8^, ^42^

A scoping review exploring women's experiences with GBV support systems across diverse contexts reveals persistent barriers shaped by the intersection of racialization, immigration status, poverty, and other systemic factors, which are often overlooked in generic support models that fail to adequately address the needs of these diverse groups.^43^

Several systematic reviews have identified multiple risk factors in low‐ and middle‐income countries that contribute to the prevalence of one of the most common forms of GBV, intimate partner violence (IPV).^44^, ^45^ Individual, relationship, community, and societal factors have been described in the literature as being associated with this type of violence.^40^

Sociodemographic characteristics such as lower levels of education, early marriage, socioeconomic vulnerabilities, and residence in rural areas have been associated with higher IPV prevalence in these settings.^44^ Adolescents and younger women are also exposed to various forms of IPV in low‐ and middle‐income countries.^46^

A systematic review estimating the global prevalence of sexual violence against children using national‐level, population‐based studies from 80 countries found that the lifetime prevalence of sexual harassment among girls was 14.1% (95% CI, 10.4%–18.8%).^47^ This evidence demonstrates that experiences of violence often begin in childhood and persist across the life course, leading to adverse health outcomes and compromising well‐being.

Moreover, evidence also indicates that a history of violence increases the likelihood of experiencing violence in adulthood.^45^, ^46^ Childhood exposure to violence was associated with increased odds of experiencing violence later in life, with estimates ranging from 1.46 in peri‐urban Ghana to 3.15 in Nepal.^45^

Partner characteristics, particularly alcohol or substance use, consistently increased the risk of IPV. For instance, in Vietnam, the odds increased from 2.97 among men who drank monthly to 3.32 among those who drank weekly and reached 7.06 among daily drinkers.^48^

Evidence regarding sociodemographic factors and their association with violence exposure is heterogeneous and varies across regions and countries.^42^ Thus, contextual specificities must be considered when implementing effective prevention measures.

The experience of violence in historically vulnerable groups must be understood through the intersections of health determinants such as socioeconomic conditions and social context factors, including public policies and access to services that address their specific needs.^42^

Additionally, stigma and cultural norms that reinforce the acceptability of violence, limited community support, and gaps in public policies hinder interventions in this area.^45^ Domestic violence tends to receive less political recognition and is often regarded as a lower priority compared with other health concerns within the healthcare sector.^9^ This highlights the need for stronger state commitment to addressing this issue.

In this sense, among the strategies, advocacy and counseling interventions have shown promising results, especially in empowering women to identify signs of violence, elaborating safety plans, facilitate access to resources according to women's needs, and assist women in identifying the most suitable responses for their specific circumstances.^16^, ^28^, ^35^

In SRH services, the psychosocial interventions for VAW survivors in addition to the routine consultations show robust outcomes enhancing safety behaviors.^3^ For pregnant and postpartum women, home visitation programs and some multifaceted counseling interventions also produced promising results.^18^

In particular, antenatal and postpartum care consultations are important settings in which systematic screening for violence can be integrated. A study conducted with 600 Brazilian pregnant and postpartum women showed that Black women (odds ratio [OR] = 1.53; 95% confidence interval [CI] 1.01–2.34; *P* = 0.048), gestational age ≤ 13 weeks (OR = 3.41; 95% CI 1.03–11.25; *P* = 0.044), and those in the postpartum period (OR = 2.81; 95% CI 1.32–5.99; *P* = 0.008) were more likely to have experienced domestic violence at some point in their lives.^49^

Routine inquiry during prenatal care and postpartum check‐ups, along with providing or referring to intervention services when needed, is essential. In this regard, healthcare pathways should be considered as a clinical practice for GBV survivors focused on the continuity of care and mitigation of health consequences, ensuring women at risk receive appropriate follow‐up at secondary and tertiary level of care.

### 5.1 Strengths and limitations

This review summarizes the knowledge on GBV interventions in healthcare settings, identifying evidence that would potentially improve the health system's response to confront this issue. The use of evidence synthesis methods contributes to informing policymakers and health system stakeholders about a spectrum of interventions, their potential benefits, and challenges in implementing them. However, some limitations must be considered.

The main limitation concerns the context in which the evidence was produced. Most of the reviews included primary studies conducted in high‐income countries. This could limit the opportunities to generalize the findings due to the challenges that LMICs face in implementing interventions that require financial and human resources.

This study was limited by the absence of a search for gray literature in the search strategy. However, a comprehensive search was used through various databases that contributed to retrieving papers from diverse sources.

Of the 24 reviews, 10 met most or all of the checklist criteria, demonstrating high quality and indicating strong methodological rigor and transparency, with ≥80% of the criteria met. However, some methodological limitations were identified in the remaining 14 reviews.

Among the reviews assessed as having moderate or low quality, some factors appeared to influence the overall rating. These included the lack of alignment between the inclusion criteria and the review question, as well as uncertainty regarding the appropriateness of the search strategy. Moreover, one review included only two databases, which might have limited the comprehensiveness of the evidence search.

In addition, seven reviews did not report a critical appraisal of the primary studies using instruments appropriate to the research question and study design. Even among those that reported conducting a critical appraisal, five did not clearly state whether the assessment was performed independently by two or more reviewers. Similarly, strategies to minimize data extraction bias and procedures for data analysis were unclear in eight reviews.

The item related to the evaluation of risk of bias in the included publications was also insufficiently addressed, largely due to the nature of the primary studies, which consisted of qualitative research. Overall, the reviews proposed valuable recommendations for practice, policy, and healthcare professionals. However, the methodological limitations described above should be considered in future replications of the studies and in the implementation of these recommendations.

## 6 CONCLUSION

The systematic reviews analyzed included studies that address interventions with a variety of tools and methods to respond to GBV victimization among at‐risk populations, particularly in sexual and reproductive services.

Most of the primary studies included screening as a first to enhance the early identification of vulnerable groups. However, implementing protocols that facilitate secondary or tertiary‐level interventions for survivors when necessary is recommended.

Further, it is crucial to implement multifaceted interventions, incorporate educational components into prevention strategies, and adopt measures by managers and policymakers that enhance the capacities of health systems. Evaluating interventions that show high potential effectiveness is essential for guiding health policy and allocating resources for women's healthcare services in LMICs. Moreover, culturally sensitive strategies based on women‐centered, diversity‐inclusive, and equity approaches must guide the implementation of GBV interventions.

## AUTHOR CONTRIBUTIONS

Design and planning: ORS, LR, EZ and FGS. Data selection and extraction: ORS, NQD, and EZ. Analysis, interpretation, writing the paper and critical review of its content: ORS, LR, EZ, NQD and FGS. Supervision: FGS. All the authors approved the final version.

## FUNDING INFORMATION

This study was financed in part by the National Council for Scientific and Technological Development – CNPq and the Department of Science and Technology of Secretariat of Science, Technology, Innovation and Health Complex of Ministry of Health of Brazil – MoH. Project number 444414/2023‐1 CNPq. Proposal Call No. 21/2023 – Transdisciplinary Studies in Public Health.

## CONFLICT OF INTEREST STATEMENT

The authors declare that they have no competing interests in performing this work.

## Supporting information


**Data S1:** Preferred Reporting Items for Systematic reviews and Meta‐Analyses extension for Scoping Reviews (PRISMA‐ScR) Checklist.

**Table S1.** Search strategy.
**Table S2.** JBI Critical Appraisal Checklist for Systematic Reviews and Research Syntheses.
**Table S3.** List of excluded studies n = 53.
**Table S4.** Implications for healthcare stakeholders, public policies and research.

## Data Availability

The datasets used and/or analyzed during the current study are available from the corresponding author on reasonable request.

## References

[1] World Health Organization . Caring for women subjected to violence: a WHO curriculum for training health‐care providers [WHO website]. 2019. Accessed February 8, 2025. https://www.who.int/publications/i/item/9789240039803

[2] World Health Organization . Violence against Women Prevalence Estimates, 2018 Global, Regional and National Prevalence Estimates for Intimate Partner Violence against Women and Global and Regional Prevalence Estimates for Non‐partner Sexual Violence against Women. World Health Organization; 2021.

[3] Lewis NV , Munas M , Colombini M , et al. Interventions in sexual and reproductive health services addressing violence against women in low‐income and middle‐income countries: a mixed‐methods systematic review. BMJ Open. 2022;12(2):e051924. 10.1136/bmjopen-2021-051924 PMC8867339 35193906

[4] Campbell JC . Health consequences of intimate partner violence. Lancet. 2002;359(9314):1331‐1336. 11965295 10.1016/S0140-6736(02)08336-8

[5] Black MC . Intimate partner violence and adverse health consequences. Am J Lifestyle Med. 2011;5(5):428‐439.

[6] Pallitto CC , García‐Moreno C , Jansen HAFM , Heise L , Ellsberg M , Watts C . Intimate partner violence, abortion, and unintended pregnancy: results from the WHO multi‐country study on women's health and domestic violence. Int J Gynaecol Obstet. 2013;120(1):3‐9. 22959631 10.1016/j.ijgo.2012.07.003

[7] Surita FG , Sánchez ODR . Routine enquiry for domestic violence during antenatal care: an opportunity to improve Women's health. Rev Bras Ginecol Obstet. 2022;44(3):211‐213. 35576935 10.1055/s-0042-1742735 PMC9948041

[8] García‐Moreno C , Hegarty K , d'Oliveira AF , Koziol‐McLain J , Colombini M , Feder G . The health‐systems response to violence against women. Lancet. 2015;385(9977):1567‐1579. 25467583 10.1016/S0140-6736(14)61837-7

[9] d'Oliveira AFPL , Pereira S , Bacchus LJ , et al. Are we asking too much of the health sector? Exploring the readiness of Brazilian primary healthcare to respond to domestic violence against women. Int J Health Policy Manag. 2022;11(7):961‐972. 33327691 10.34172/ijhpm.2020.237 PMC9808197

[10] World Health Organization . Responding to Intimate Partner Violence and Sexual Violence Against Women: WHO Clinical and Policy Guidelines. World Health Organization; 2013. 24354041

[11] Aromataris E , Fernandez R , Godfrey C , Holly C , Khalil H , Tungpunkom P . Umbrella Reviews (2020). In: Aromataris E , Lockwood C , Porritt K , Pilla B , Jordan Z , eds. JBI Manual for Evidence Synthesis. JBI; 2024. Accessed February 8, 2025. https://synthesismanual.jbi.global. 10.46658/JBIMES-24-08

[12] Langlois ÉV , Daniels K , Akl EA , eds. Evidence Synthesis for Health Policy and Systems: A Methods Guide. World Health Organization; 2018. 33877749

[13] Brazil. Ministry of Health. Secretariat of Science, Technology, Innovation, and Strategic Health Supplies. Department of Science and Technology . Methodological Guideline: Evidence Synthesis for Policies. Ministry of Health; 2020.

[14] World Health Organization . Adolescent Health [WHO website]. 2025. Accessed February 8, 2025. https://www.who.int/health‐topics/adolescent‐health#tab=tab_1.2025

[15] Prosman GJ , Lo Fo Wong SH , van der Wouden JC , Lagro‐Janssen AL . Effectiveness of home visiting in reducing partner violence for families experiencing abuse: a systematic review. Fam Pract. 2015;32(3):247‐256. 25947931 10.1093/fampra/cmu091

[16] Sapkota D , Baird K , Saito A , Anderson D . Interventions for reducing and/or controlling domestic violence among pregnant women in low‐ and middle‐income countries: a systematic review. Syst Rev. 2019;8(1):79. 30940204 10.1186/s13643-019-0998-4 PMC6889323

[17] Linde DS , Bakiewicz A , Normann AK , Hansen NB , Lundh A , Rasch V . Intimate partner violence and electronic health interventions: systematic review and meta‐analysis of randomized trials. J Med Internet Res. 2020;22(12):e22361. 33306030 10.2196/22361 PMC7762681

[18] Van Parys AS , Verhamme A , Temmerman M , Verstraelen H . Intimate partner violence and pregnancy: a systematic review of interventions. PLoS One. 2014;9(1):e85084. 24482679 10.1371/journal.pone.0085084 PMC3901658

[19] Hussain N , Sprague S , Madden K , Hussain FN , Pindiprolu B , Bhandari M . A comparison of the types of screening tool administration methods used for the detection of intimate partner violence: a systematic review and meta‐analysis. Trauma Violence Abuse. 2015;16(1):60‐69. 24343478 10.1177/1524838013515759

[20] Jahanfar S , Howard LM , Medley N . Interventions for preventing or reducing domestic violence against pregnant women. Cochrane Database Syst Rev. 2014;2014(11):CD009414. 25390767 10.1002/14651858.CD009414.pub3 PMC7104547

[21] O'Doherty L , Hegarty K , Ramsay J , Davidson LL , Feder G , Taft A . Screening women for intimate partner violence in healthcare settings. Cochrane Database Syst Rev. 2015;2015(7):CD007007. 26200817 10.1002/14651858.CD007007.pub3 PMC6599831

[22] Adams C , Hooker L , Taft A . A systematic review and qualitative meta‐synthesis of the roles of home‐visiting nurses working with women experiencing family violence. J Adv Nurs. 2023;79(4):1189‐1210. 35285982 10.1111/jan.15224

[23] El Morr C , Layal M . Effectiveness of ICT‐based intimate partner violence interventions: a systematic review. BMC Public Health. 2020;20(1):1372. 32894115 10.1186/s12889-020-09408-8 PMC7476255

[24] Sabloak T , Ryan I , Nahi S , Eucalitto P , Simon MA , Premkumar A . Intimate partner violence detected during abortion‐related visits: a systematic review of screenings and interventions. Am J Perinatol. 2024;41(12):1697‐1705. 38365213 10.1055/s-0044-1779746

[25] Bair‐Merritt MH , Lewis‐O'Connor A , Goel S , et al. Primary care‐based interventions for intimate partner violence: a systematic review. Am J Prev Med. 2014;46(2):188‐194. 24439354 10.1016/j.amepre.2013.10.001

[26] Teichman AL , Bonne S , Rattan R , et al. Screening and intervention for intimate partner violence at trauma centers and emergency departments: an evidence‐based systematic review from the eastern Association for the Surgery of Trauma. Trauma Surg Acute Care Open. 2023;8(1):e001041. 36967863 10.1136/tsaco-2022-001041 PMC10030790

[27] Miller CJ , Adjognon OL , Brady JE , Dichter ME , Iverson KM . Screening for intimate partner violence in healthcare settings: an implementation‐oriented systematic review. Implement Res Pract. 2021;2:263348952110398. 10.1177/26334895211039894 PMC9881185 36712586

[28] Trabold N . Screening for intimate partner violence within a health care setting: a systematic review of the literature. Soc Work Health Care. 2007;45(1):1‐18. 10.1300/J010v45n01_01 17804344

[29] Howell KH , Miller‐Graff LE , Hasselle AJ , Scrafford KE . The unique needs of pregnant, violence‐exposed women: a systematic review of current interventions and directions for translational research. Aggress Violent Behav. 2017;34:128‐138.

[30] Tomsett B , Álvarez‐Rodríguez J , Sherriff N , Edelman N , Gatuguta A . Tools for the identification of victims of domestic abuse and modern slavery in remote services: a systematic review. J Health Serv Res Policy. 2025;30(1):63‐76. 38849123 10.1177/13558196241257864 PMC11673303

[31] Ahmad F , Mahmood S . What do we know about interactive computer‐assisted screening for intimate partner violence and control in clinical settings? A systematic review. Ital J Public Health. 2010;7(2):48‐58.

[32] Nelson HD , Bougatsos C , Blazina I . Screening women for intimate partner violence: a systematic review to update the U.S. Preventive Services Task Force Recommendation. Ann Intern Med. 2012;156(11):796. 22565034 10.7326/0003-4819-156-11-201206050-00447

[33] Daley D , McCauley M , Van Den Broek N . Interventions for women who report domestic violence during and after pregnancy in low‐ and middle‐income countries: a systematic literature review. BMC Pregnancy Childbirth. 2020;20(1):141. 32138721 10.1186/s12884-020-2819-0 PMC7059681

[34] O'Reilly R , Beale B , Gillies D . Screening and intervention for domestic violence during pregnancy care: a systematic review. Trauma Violence Abuse. 2010;11(4):190‐201. 20688785 10.1177/1524838010378298

[35] Kataoka Y , Yaju Y , Eto H , Matsumoto N , Horiuchi S . Screening of domestic violence against women in the perinatal setting: a systematic review. Jpn J Nurs Sci. 2004;1(2):77‐86.

[36] Basheer MB , Bell R , Boyle AA . Systematic review of the effectiveness of advocacy interventions for adult victims of domestic violence within an emergency department setting. Cureus. 2022;14(6):e25599. 35784992 10.7759/cureus.25599 PMC9249019

[37] Anderson EJ , Krause KC , Meyer Krause C , et al. Web‐based and mHealth interventions for intimate partner violence victimization prevention: a systematic review. Trauma Violence Abuse. 2021;22(4):870‐884. 31742475 10.1177/1524838019888889

[38] Sikder SS , Ghoshal R , Bhate‐Deosthali P , Jaishwal C , Roy N . Mapping the health systems response to violence against women: key learnings from five LMIC settings (2015‐2020). BMC Womens Health. 2021;21(1):360. 34629077 10.1186/s12905-021-01499-8 PMC8504083

[39] Recommendations for Preventive Services for Women Final Report to the U.S. Department of Health and Human Services, Health Resources & Services Administration December 2016. American College of Obstetricians and Gynecologists; 2017.

[40] Chisholm CA , Bullock L , Ferguson JEJ 2nd . Intimate partner violence and pregnancy: screening and intervention. Am J Obstet Gynecol. 2017;217(2):145‐149. 28551447 10.1016/j.ajog.2017.05.043

[41] Labre DM , Del Risco Sánchez O , Monteiro I , Freitas‐Jesus JV , Surita FG . Addressing domestic violence by obstetric and gynecological resident doctors: pre‐post intervention study. BMC Med Educ. 2025;25(1):1405. 41077576 10.1186/s12909-025-08010-z PMC12516890

[42] Sánchez ODR , Rodrigues L , Zambrano E , Borrego NG , Surita FG . Gender‐based violence in Brazil: a scoping review of risk factors and associated health consequences. Ciênc Saúde Colet. 2024;31(2):e17332024. [cited 2025 Dec 23]. Available from: http://cienciaesaudecoletiva.com.br/artigos/violencia‐de‐genero‐no‐brasil‐revisao‐de‐escopo‐sobre‐fatores‐de‐risco‐e‐agravos‐a‐saude/19451?id=19451&id=19451 10.1590/1413-81232026312.17332024 41779585

[43] Essue BM , Chadambuka C , Perez‐Brumer A , et al. Women's experiences of gender‐based violence supports through an intersectional lens: a global scoping review. BMJ Public Health. 2025;3(1):e001405. 40017981 10.1136/bmjph-2024-001405 PMC11816106

[44] Gunarathne L , Bhowmik J , Apputhurai P , Nedeljkovic M . Factors and consequences associated with intimate partner violence against women in low‐ and middle‐income countries: a systematic review. PLoS One. 2023;18(11):e0293295. 37939106 10.1371/journal.pone.0293295 PMC10631698

[45] Ghoshal R , Douard AC , Sikder S , Roy N , Saulnier D . Risk and protective factors for IPV in low‐ and middle‐income countries: a systematic review. J Aggress Maltreat Trauma. 2023;32(4):505‐522.

[46] Abramsky T , Watts CH , Garcia‐Moreno C , et al. What factors are associated with recent intimate partner violence? Findings from the WHO multi‐country study on women's health and domestic violence. BMC Public Health. 2011;11(16):109. 21324186 10.1186/1471-2458-11-109 PMC3049145

[47] Piolanti A , Schmid IE , Fiderer FJ , Ward CL , Stöckl H , Foran HM . Global prevalence of sexual violence against children: a systematic review and meta‐analysis. JAMA Pediatr. 2025;179(3):264‐272. 39804632 10.1001/jamapediatrics.2024.5326 PMC11877167

[48] Jansen HAFM , Nguyen TVN , Hoang TA . Exploring risk factors associated with intimate partner violence in Vietnam: results from a cross‐sectional national survey. Int J Public Health. 2016;61(8):923‐934. 27554794 10.1007/s00038-016-0879-8

[49] Sánchez ODR , Tanaka Zambrano E , Dantas‐Silva A , et al. Domestic violence: a cross‐sectional study among pregnant and postpartum women. J Adv Nurs. 2023;79(4):1525‐1539. 35855530 10.1111/jan.15375

