# Supplementary material for: Strategies for preventing gender‐based violence in healthcare services: Evidence synthesis for health policy
Source: Int J Gynaecol Obstet. 2026 Mar 23;174(3):1235–53. doi: 10.1002/ijgo.70934 (PMC13505212; doi:10.1002/ijgo.70934)
Supplement: Supplementary file 1 [file IJGO-174-1235-s002.docx]

**Table S1.** Search strategy

| **Database** | **Search strategy** |
| --- | --- |
| **PUBMED** | ((((((Women[MeSH Terms]) OR (Women[Title/Abstract] OR Girls[Title/Abstract] OR Girl[Title/Abstract] OR Woman[Title/Abstract] OR "Women Groups"[Title/Abstract])) OR (Female[MeSH Terms])) OR (Female[Title/Abstract] OR Females[Title/Abstract])) AND (((((((Domestic Violence[MeSH Terms]) OR ("Domestic Violence"[Title/Abstract] OR "Violence, Domestic"[Title/Abstract] OR "Family Violence"[Title/Abstract] OR "Violence, Family"[Title/Abstract])) OR ("Violence Against Women"[Title/Abstract])) OR (Gender-Based Violence[MeSH Terms])) OR ("Gender-Based Violence"[Title/Abstract] OR "Gender Based Violence"[Title/Abstract] OR "Violence, Gender-Based"[Title/Abstract] OR "Dowry Death"[Title/Abstract] OR "Dowry Deaths"[Title/Abstract])) OR (Intimate Partner Violence[MeSH Terms])) OR ("Intimate Partner Violence"[Title/Abstract] OR "Partner Violence, Intimate"[Title/Abstract] OR "Violence, Intimate Partner"[Title/Abstract] OR "Intimate Partner Abuse"[Title/Abstract] OR "Abuse, Intimate Partner"[Title/Abstract] OR "Dating Violence"[Title/Abstract] OR "Violence, Dating"[Title/Abstract]))) AND (((((Mass Screening[MeSH Terms]) OR ("Mass Screening"[Title/Abstract] OR "Mass Screenings"[Title/Abstract] OR Screen*[Title/Abstract])) OR (Interventions[Title/Abstract] OR Intervention[Title/Abstract])) OR (Precenting[Title/Abstract] OR Prevent[Title/Abstract])) OR ("precautionary action"[Title/Abstract] OR "preventive action"[Title/Abstract] OR "preventive measure"[Title/Abstract] OR prevention[Title/Abstract]))) AND ((((((((Systematic Review[Publication Type]) OR ("Systematic Review"[Title/Abstract] OR "Review, Systematic"[Title/Abstract])) OR ("Meta-Analysis"[Publication Type])) OR ("Meta-Analysis"[Title/Abstract])) OR ("analysis, meta"[Title/Abstract] OR "meta analysis"[Title/Abstract] OR metaanalysis[Title/Abstract])) OR (Network Meta-Analysis[Publication Type])) OR ("Network Meta-Analysis"[Title/Abstract] OR "Network Meta Analysis"[Title/Abstract] OR "Network Meta-Analyses"[Title/Abstract] OR "Multiple Treatment Comparison Meta-Analysis"[Title/Abstract] OR "Multiple Treatment Comparison Meta Analysis"[Title/Abstract] OR "Mixed Treatment Meta-Analysis"[Title/Abstract] OR "Mixed Treatment Meta Analysis"[Title/Abstract] OR "Mixed Treatment Meta-Analyses"[Title/Abstract])) OR ("network metaanalyses"[Title/Abstract] OR "network metaanalysis"[Title/Abstract] OR "network meta-analysis"[Title/Abstract])) |
| **Health Systems Evidence** | (Women OR Female) AND ("Domestic Violence" OR "Violence Against Women" OR "Gender-Based Violence" OR "Intimate Partner Violence") AND ("Mass Screening" OR Interventions OR Intervention OR Prevention OR Precenting OR Prevent) AND ("Systematic Review" OR "Meta-Analysis" OR "Network Meta-Analysis) |
| **BVS / BIREME**  MEDLINE (488)  LILACS (21)  IBECS (7)  BDENF - Enfermagem (4)  Index Psicologia - Periódicos (3)  BIGG - guias GRADE (1)  CUMED (1)  PIE (1) | (women OR girls OR girl OR woman OR "Women Groups") OR (female OR females ) AND (("Domestic Violence" OR "Violence, Domestic" OR "Family Violence" OR "Violence, Family") OR ("Violence Against Women") OR ("Gender-Based Violence" OR "Gender Based Violence" OR "Violence, Gender-Based" OR "Dowry Death" OR "Dowry Deaths") OR ("Intimate Partner Violence" OR "Partner Violence, Intimate" OR "Violence, Intimate Partner" OR "Intimate Partner Abuse" OR "Abuse, Intimate Partner" OR "Dating Violence" OR "Violence, Dating")) AND (("Mass Screening" OR "Mass Screenings" OR screen*) OR (interventions OR intervention) OR (precenting OR prevent) OR ("precautionary action" OR "preventive action" OR "preventive measure" OR prevention)) AND (("Systematic Review" OR "Review, Systematic") OR ("Meta-Analysis") OR ("analysis, meta" OR "meta analysis" OR metaanalysis) OR ("Network Meta-Analysis" OR "Network Meta Analysis" OR "Network Meta-Analyses" OR "Multiple Treatment Comparison Meta-Analysis" OR "Multiple Treatment Comparison Meta Analysis" OR "Mixed Treatment Meta-Analysis" OR "Mixed Treatment Meta Analysis" OR "Mixed Treatment Meta-Analyses") OR ("network metaanalyses" OR "network metaanalysis" OR "network meta-analysis")) |
| **CINAHL - The Cumulative Index to Nursing and Allied Health Literature** | (MM "Women+") OR TI ( Women OR Girls OR Girl OR Woman OR "Women Groups" ) OR AB ( Women OR Girls OR Girl OR Woman OR "Women Groups" ) OR (MM "Female") OR TI ( Female OR Females ) OR AB ( Female OR Females ) AND (MM "Domestic Violence+") OR TI ( "Domestic Violence" OR "Violence, Domestic" OR "Family Violence" OR "Violence, Family" ) OR AB ( "Domestic Violence" OR "Violence, Domestic" OR "Family Violence" OR "Violence, Family" ) OR TI "Violence Against Women" OR AB "Violence Against Women" OR (MM "Gender-Based Violence") OR TI ( "Gender-Based Violence" OR "Gender Based Violence" OR "Violence, Gender-Based" OR "Dowry Death" OR "Dowry Deaths" ) OR AB ( "Gender-Based Violence" OR "Gender Based Violence" OR "Violence, Gender-Based" OR "Dowry Death" OR "Dowry Deaths" ) OR (MM "Intimate Partner Violence") OR TI ( "Intimate Partner Violence" OR "Partner Violence, Intimate" OR "Violence, Intimate Partner" OR "Intimate Partner Abuse" OR "Abuse, Intimate Partner" OR "Dating Violence" OR "Violence, Dating" ) OR AB ( "Intimate Partner Violence" OR "Partner Violence, Intimate" OR "Violence, Intimate Partner" OR "Intimate Partner Abuse" OR "Abuse, Intimate Partner" OR "Dating Violence" OR "Violence, Dating" ) AND TI ( "Mass Screening" OR "Mass Screenings" OR Screen* ) OR AB ( "Mass Screening" OR "Mass Screenings" OR Screen* ) OR TI ( Interventions OR Intervention ) OR AB ( Interventions OR Intervention ) OR TI ( Precenting OR Prevent ) OR AB ( Precenting OR Prevent ) OR TI ( "precautionary action" OR "preventive action" OR "preventive measure" OR prevention ) OR AB ( "precautionary action" OR "preventive action" OR "preventive measure" OR prevention ) AND (MM "Systematic Review") OR TI ( "Systematic Review" OR "Review, Systematic" ) OR AB ( "Systematic Review" OR "Review, Systematic" ) OR (MM "Meta Analysis") OR TI "Meta-Analysis" OR AB "Meta-Analysis" OR TI ( "analysis, meta" OR "meta analysis" OR metaanalysis ) OR AB ( "analysis, meta" OR "meta analysis" OR metaanalysis ) OR TI ( "Network Meta-Analysis" OR "Network Meta Analysis" OR "Network Meta-Analyses" OR "Multiple Treatment Comparison Meta-Analysis" OR "Multiple Treatment Comparison Meta Analysis" OR "Mixed Treatment Meta-Analysis" OR "Mixed Treatment Meta Analysis" OR "Mixed Treatment Meta-Analyses" ) OR AB ( "Network Meta-Analysis" OR "Network Meta Analysis" OR "Network Meta-Analyses" OR "Multiple Treatment Comparison Meta-Analysis" OR "Multiple Treatment Comparison Meta Analysis" OR "Mixed Treatment Meta-Analysis" OR "Mixed Treatment Meta Analysis" OR "Mixed Treatment Meta-Analyses" ) OR TI ( "network metaanalyses" OR "network metaanalysis" OR "network meta-analysis" ) OR AB ( "network metaanalyses" OR "network metaanalysis" OR "network meta-analysis" ) |
| **SCOPUS** | ( ( TITLE-ABS-KEY ( women OR girls OR girl OR woman OR "Women Groups" ) OR TITLE-ABS-KEY ( female OR females ) ) ) AND ( ( TITLE-ABS-KEY ( "Domestic Violence" OR "Violence, Domestic" OR "Family Violence" OR "Violence, Family" ) OR TITLE-ABS-KEY ( "Violence Against Women" ) OR TITLE-ABS-KEY ( "Gender-Based Violence" OR "Gender Based Violence" OR "Violence, Gender-Based" OR "Dowry Death" OR "Dowry Deaths" ) OR TITLE-ABS-KEY ( "Intimate Partner Violence" OR "Partner Violence, Intimate" OR "Violence, Intimate Partner" OR "Intimate Partner Abuse" OR "Abuse, Intimate Partner" OR "Dating Violence" OR "Violence, Dating" ) ) ) AND ( ( TITLE-ABS-KEY ( "Mass Screening" OR "Mass Screenings" OR screen* ) OR TITLE-ABS-KEY ( interventions OR intervention ) OR TITLE-ABS-KEY ( precenting OR prevent ) OR TITLE-ABS-KEY ( "precautionary action" OR "preventive action" OR "preventive measure" OR prevention ) ) ) AND ( ( TITLE-ABS-KEY ( "Systematic Review" OR "Review, Systematic" ) OR TITLE-ABS-KEY ( "Meta-Analysis" ) OR TITLE-ABS-KEY ( "analysis, meta" OR "meta analysis" OR metaanalysis ) OR TITLE-ABS-KEY ( "Network Meta-Analysis" OR "Network Meta Analysis" OR "Network Meta-Analyses" OR "Multiple Treatment Comparison Meta-Analysis" OR "Multiple Treatment Comparison Meta Analysis" OR "Mixed Treatment Meta-Analysis" OR "Mixed Treatment Meta Analysis" OR "Mixed Treatment Meta-Analyses" ) OR TITLE-ABS-KEY ( "network metaanalyses" OR "network metaanalysis" OR "network meta-analysis" ) ) ) |
| **WEB OF SCIENCE** | Women OR Girls OR Girl OR Woman OR "Women Groups" (Topic) or Female OR Females (Topic) and Preprint Citation Index (Exclude – Database) AND "Domestic Violence" OR "Violence, Domestic" OR "Family Violence" OR "Violence, Family" (Topic) or "Violence Against Women" (Topic) or "Gender-Based Violence" OR "Gender Based Violence" OR "Violence, Gender-Based" OR "Dowry Death" OR "Dowry Deaths" (Topic) or "Intimate Partner Violence" OR "Partner Violence, Intimate" OR "Violence, Intimate Partner" OR "Intimate Partner Abuse" OR "Abuse, Intimate Partner" OR "Dating Violence" OR "Violence, Dating" (Topic) and Preprint Citation Index (Exclude – Database) AND "Mass Screening" OR "Mass Screenings" OR Screen* (Topic) or Interventions OR Intervention (Topic) or Precenting OR Prevent (Topic) or "precautionary action" OR "preventive action" OR "preventive measure" OR prevention (Topic) and Preprint Citation Index (Exclude – Database) AND "Systematic Review" OR "Review, Systematic" (Topic) or "Meta-Analysis" (Topic) or "analysis, meta" OR "meta analysis" OR metaanalysis (Topic) or "Network Meta-Analysis" OR "Network Meta Analysis" OR "Network Meta-Analyses" OR "Multiple Treatment Comparison Meta-Analysis" OR "Multiple Treatment Comparison Meta Analysis" OR "Mixed Treatment Meta-Analysis" OR "Mixed Treatment Meta Analysis" OR "Mixed Treatment Meta-Analyses" (Topic) or "network metaanalyses" OR "network metaanalysis" OR "network meta-analysis" (Topic) and Preprint Citation Index (Exclude – Database) |
| **EMBASE** | ('female'/syn OR girls:ti,ab,kw OR girl:ti,ab,kw OR woman:ti,ab,kw OR 'women groups':ti,ab,kw) AND ('domestic violence'/syn OR 'violence against women':ti,ab,kw OR 'gender based violence'/syn OR 'partner violence'/syn) AND ('mass screening'/syn OR screen*:ti,ab,kw OR interventions:ti,ab,kw OR intervention:ti,ab,kw OR precenting:ti,ab,kw OR prevent:ti,ab,kw OR 'prevention'/syn) AND (systematic AND 'review'/syn OR 'meta analysis'/syn OR 'network meta-analysis'/syn) AND ([embase]/lim NOT ([embase]/lim AND [medline]/lim) OR ([medline]/lim NOT ([embase]/lim AND [medline]/lim) NOT ([embase classic]/lim AND [medline]/lim))) |
| **COCHRANE LIBRARY** | MeSH descriptor: [Women] explode all trees OR (Women OR Girls OR Girl OR Woman OR "Women Groups"):ti,ab,kw OR MeSH descriptor: [Female] in all MeSH products OR (Female OR Females):ti,ab,kw AND MeSH descriptor: [Domestic Violence] explode all trees OR ("Domestic Violence" OR "Violence, Domestic" OR "Family Violence" OR "Violence, Family"):ti,ab,kw OR ("Violence Against Women"):ti,ab,kw OR MeSH descriptor: [Gender-Based Violence] explode all trees OR ("Gender-Based Violence" OR "Gender Based Violence" OR "Violence, Gender-Based" OR "Dowry Death" OR "Dowry Deaths"):ti,ab,kw OR MeSH descriptor: [Intimate Partner Violence] explode all trees OR ("Intimate Partner Violence" OR "Partner Violence, Intimate" OR "Violence, Intimate Partner" OR "Intimate Partner Abuse" OR "Abuse, Intimate Partner" OR "Dating Violence" OR "Violence, Dating"):ti,ab,kw AND MeSH descriptor: [Mass Screening] explode all trees OR ("Mass Screening" OR "Mass Screenings" OR Screen*):ti,ab,kw OR (Interventions OR Intervention):ti,ab,kw OR (Precenting OR Prevent):ti,ab,kw OR ("precautionary action" OR "preventive action" OR "preventive measure" OR prevention):ti,ab,kw AND ("Systematic Review" OR "Review, Systematic"):ti,ab,kw OR ("Meta-Analysis"):ti,ab,kw OR ("analysis, meta" OR "meta analysis" OR metaanalysis):ti,ab,kw OR ("Network Meta-Analysis" OR "Network Meta Analysis" OR "Network Meta-Analyses" OR "Multiple Treatment Comparison Meta-Analysis" OR "Multiple Treatment Comparison Meta Analysis" OR "Mixed Treatment Meta-Analysis" OR "Mixed Treatment Meta Analysis" OR "Mixed Treatment Meta-Analyses"):ti,ab,kw OR ("network metaanalyses" OR "network metaanalysis" OR "network meta-analysis"):ti,ab,kw |

**Table S2.** JBI Critical Appraisal Checklist for Systematic Reviews and Research Syntheses

| **#** | **Author** | **Title** | **Q1** | **Q2** | **Q3** | **Q4** | **Q5** | **Q6** | **Q7** | **Q8** | **Q9** | **Q10** | **Q11** | **Score** |
| --- | --- | --- | --- | --- | --- | --- | --- | --- | --- | --- | --- | --- | --- | --- |
| 1 | Hussain et al., 2015 | A comparison of the types of screening tool administration methods used for the detection of intimate partner violence: a systematic review and meta-analysis. | Y | Y | Y | Y | Y | Y | Y | Y | N | Y | Y | 10Y 1N |
| 2 | Adams et al., 2022 | A systematic review and qualitative meta-synthesis of the roles of home-visiting nurses working with women experiencing family violence. | Y | Y | U | Y | Y | Y | U | Y | N/A | Y | Y | 8Y 1N/A 2U |
| 3 | Prosman et al., 2015 | Effectiveness of home visiting in reducing partner violence for families experiencing abuse: a systematic review. | Y | Y | Y | Y | Y | Y | U | Y | N/A | Y | Y | 9Y 1N/A 1U |
| 4 | Jahanfar et al., 2014 | Interventions for preventing or reducing domestic violence against pregnant women. | Y | Y | Y | Y | Y | Y | Y | Y | Y | Y | Y | 11Y |
| 5 | Sapkota et al., 2019 | Interventions for reducing and/or controlling domestic violence among pregnant women in low- and middle-income countries: a systematic review. | Y | Y | Y | Y | Y | Y | Y | Y | N | Y | Y | 10Y 1N |
| 6 | Daley et al., 2020 | Interventions for women who report domestic violence during and after pregnancy in low- and middle-income countries: a systematic literature review. | Y | Y | Y | Y | Y | U | Y | Y | Y | Y | Y | 10Y 1U |
| 7 | Lewis et al., 2022 | Interventions in sexual and reproductive health services addressing violence against women in low-income and middle-income countries: a mixed-methods systematic review. | Y | Y | U | Y | Y | U | Y | Y | N/A | Y | Y | 8Y 1N/A 2U |
| 8 | Linde et al., 2020 | Intimate Partner Violence and Electronic Health Interventions: Systematic Review and Meta-Analysis of Randomized Trials. | Y | Y | Y | Y | Y | Y | Y | Y | N | Y | Y | 10Y 1N |
| 9 | Van Parys et al. 2014 | Intimate partner violence and pregnancy: a systematic review of interventions. | Y | Y | Y | Y | Y | Y | Y | Y | N/A | N | Y | 9Y 1N 1NA |
| 10 | Sabloak et al., 2024 | Intimate Partner Violence Detected during Abortion-Related Visits: A Systematic Review of Screenings and Interventions. | Y | Y | Y | Y | Y | U | U | U | N/A | Y | Y | 7Y 3U 1NA |
| 11 | Bair-Merritt et al., 2014 | Primary care-based interventions for intimate partner violence: a systematic review. | Y | Y | N | Y | N | N | Y | U | N/A | Y | Y | 6Y 3N 1U 1NA |
| 12 | O'Reilly et al., 2010 | Screening and intervention for domestic violence during pregnancy care: a systematic review. | Y | Y | Y | Y | N | N | Y | Y | N/A | Y | Y | 8Y 2N 1NA |
| 13 | Teichman et al., 2023 | Screening and intervention for intimate partner violence at trauma centers and emergency departments: an evidence-based systematic review from the Eastern Association for the Surgery of Trauma | Y | Y | Y | Y | Y | Y | Y | Y | N/A | Y | Y | 10Y 1NA |
| 14 | Miller et al., 2021 | Screening for intimate partner violence in healthcare settings: An implementation-oriented systematic review. | Y | Y | Y | Y | N | N | Y | Y | N/A | Y | Y | 8Y 2N 1NA |
| 15 | Trabold, 2007 | Screening for intimate partner violence within a health care setting: a systematic review of the literature. | Y | Y | N | Y | N | N | U | U | N/A | Y | Y | 5Y 3N 2U 1NA |
| 16 | Kataoka, et al., 2004 | Screening of domestic violence against women in the perinatal setting: a systematic review. | Y | U | Y | Y | Y | Y | U | U | N/A | Y | Y | 7Y 3U 1NA |
| 17 | Nelson et al., 2012 | Screening women for intimate partner violence: a systematic review to update the U.S. Preventive Services Task Force recommendation. | Y | Y | U | Y | Y | Y | Y | U | N/A | U | Y | 7Y 3U 1NA |
| 18 | Basheer et al., 2022 | Systematic review of the effectiveness of advocacy interventions for adult victims of domestic violence within an emergency department setting | Y | U | Y | Y | Y | U | N | U | N/A | Y | Y | 6Y 1N 3U 1NA |
| 19 | Howell et al., 2017 | The unique needs of pregnant, violence-exposed women: A systematic review of current interventions and directions for translational research. | Y | Y | Y | Y | N | N | U | U | N/A | Y | Y | 6Y 2N 2U 1NA |
| 20 | Tomsett et al., 2024 | Tools for the identification of victims of domestic abuse and modern slavery in remote services: A systematic review. | Y | Y | Y | Y | Y | U | U | Y | N/A | Y | Y | 8Y 2U 1NA |
| 21 | Anderson et al., 2021 | Web-Based and mHealth Interventions for Intimate Partner Violence Victimization Prevention: A Systematic Review | Y | Y | Y | Y | Y | Y | Y | Y | N/A | Y | Y | 10Y 1NA |
| 22 | Ahmad & Mahmood, 2010 | What do we know about interactive computer-assisted screening for intimate partner violence and control in clinical settings? A systematic review | Y | Y | Y | N | N | N | U | Y | N/A | Y | Y | 6Y 3N 1U 1NA |
| 23 | O'Doherty et al., 2015 | Screening women for intimate partner violence in health care settings | Y | Y | Y | Y | Y | Y | Y | Y | Y | Y | Y | 10Y |
| 24 | El Morr & Layal et al., 2020 | Effectiveness of ICT-based intimate partner violence interventions: a systematic review. | Y | N | Y | Y | N | N | Y | U | N/A | Y | Y | 6Y 3N 1U 1NA |

Y=Yes/N=Not/U=Unclear/NA=Not applicable

JBI Critical Appraisal Checklist for Systematic Reviews and Research Syntheses [11] questions

1. Is the review question clearly and explicitly stated?
2. Were the inclusion criteria appropriate for the review question?
3. Was the search strategy appropriate?
4. Were the sources and resources used to search for studies adequate?
5. Were the criteria for appraising studies appropriate?
6. Was critical appraisal conducted by two or more reviewers independently?
7. Were there methods to minimize errors in data extraction?
8. Were the methods used to combine studies appropriate?
9. Was the likelihood of publication bias assessed?
10. Were recommendations for policy and/or practice supported by the reported data?
11. Were the specific directives for new research appropriate?

**Table S3.** List of excluded studies n=53

| **‍No** | **Reports excluded** | **Reason** |
| --- | --- | --- |
| ‍‍1 | ‍Duffy L, Adams J, Sibbritt D, Loxton D. Complementary and alternative medicine for victims of intimate partner abuse: a systematic review of use and efficacy. *Evid Based Complement Alternat Med*. 2014;2014:963967. | Type of intervention (focus on psychological/therapeutic interventions) |
| ‍‍2 | ‍Micklitz HM, Glass CM, Bengel J, Sander LB. Efficacy of Psychosocial Interventions for Survivors of Intimate Partner Violence: A Systematic Review and Meta-Analysis. *Trauma Violence Abuse.* 2024 Apr;25(2):1000-1017. | Type of intervention (focus on psychosocial interventions) |
| ‍‍3 | ‍Sabri B, Gielen A. Integrated Multicomponent Interventions for Safety and Health Risks Among Black Female Survivors of Violence: A Systematic Review*. Trauma Violence Abuse*. 2019 Dec;20(5):720-731. | Type of intervention (multicomponent intervention) |
| ‍‍4 | ‍Mootz JJ, Fennig M, Giusto A, Mumey A, Greene CM, Wainberg ML. Interventions addressing family violence and mental illness or substance use in low- and middle-income countries: A systematic review. *Glob Ment Health (Camb).* 2023 Oct 18;10:e71. | Type of intervention (multicomponent intervention) |
| ‍‍5 | ‍Marshall KJ, Fowler DN, Walters ML, Doreson AB. Interventions that Address Intimate Partner Violence and HIV Among Women: A Systematic Review. AIDS Behav. 2018 Oct;22(10):3244-3263. | Type of intervention (multicomponent intervention) |
| ‍‍6 | Camacho Soto JN, Exner-Cortens D, McMorris C, Madigan S. Secondary and Tertiary Prevention for Adolescent Dating Violence: A Systematic Review. *Trauma Violence Abuse.* 2024 Dec;25(5):3938-3950. | Type of intervention (focus on psychosocial interventions) |
| ‍‍7 | ‍Kraft JM, Wilkins KG, Morales GJ, Widyono M, Middlestadt SE. An evidence review of gender-integrated interventions in reproductive and maternal-child health. *J Health Commun.* 2014;19 Suppl 1(sup1):122-41. | Type of intervention (focus on reproductive and maternal-child health behaviors) |
| ‍‍8 | ‍van IJzendoorn MH, Bakermans-Kranenburg MJ, Coughlan B, Reijman S. Annual Research Review: Umbrella synthesis of meta-analyses on child maltreatment antecedents and interventions: differential susceptibility perspective on risk and resilience*. J Child Psychol Psychiatry.* 2020 Mar;61(3):272-290. | Type of intervention (focus on child maltreatment interventions) |
| ‍‍9 | ‍Hetrick SE, Purcell R, Garner B, Parslow R. Combined pharmacotherapy and psychological therapies for post traumatic stress disorder (PTSD). *Cochrane Database Syst Rev*. 2010 Jul 7;2010(7):CD007316. | Type of intervention (focus on psychological/therapeutic interventions ) |
| ‍‍10 | ‍Ozcevik Subasi D, Akca Sumengen A, Semerci R, Cakir GN. Effectiveness of school-based interventions on child sexual abuse knowledge in children with disabilities: Systematic review and meta-analysis. J Pediatr Nurs. 2024 Sep-Oct;78:e90-e101. | Type of intervention (school-based interventions) |
| ‍‍11 | ‍Andermann A, Mott S, Mathew CM, Kendall C, Mendonca O, Harriott D, McLellan A, Riddle A, Saad A, Iqbal W, Magwood O, Pottie K. Evidence-informed interventions and best practices for supporting women experiencing or at risk of homelessness: a scoping review with gender and equity analysis. *Health Promot Chronic Dis Prev Can*. 2021 Jan;41(1):1-13. | Type of intervention (focus on homelessness interventions) |
| ‍‍12 | ‍Orton L, Pennington A, Nayak S, Sowden A, White M, Whitehead M. Group-based microfinance for collective empowerment: a systematic review of health impacts. *Bull World Health Organ.* 2016 Sep 1;94(9):694-704A. | Type of intervention (microfinance intervention) |
| ‍‍13 | ‍Choi YJ, An S. Interventions to improve responses of helping professionals to intimate partner violence: a quick scoping review. *Res Soc Work Pract*. 2015;26(1):101-27. | Type of intervention (focus on healthcare professionals’ perspectives) |
| ‍‍14 | ‍Mason-Jones AJ, Sinclair D, Mathews C, Kagee A, Hillman A, Lombard C. School-based interventions for preventing HIV, sexually transmitted infections, and pregnancy in adolescents. *Cochrane Database Syst Rev*. 2016 Nov 8;11(11):CD006417. | Type of intervention (school-based interventions) |
| 15 | ‍Farmer C, Shaw N, Rizzo AJ, Orr N, Chollet A, Hagell A, Rigby E, Young H, Berry V, Bonell C, Melendez-Torres GJ. School-Based Interventions to Prevent Dating and Relationship Violence and Gender-Based Violence: Systematic Review and Network Meta-Analysis. *Am J Public Health.* 2023 Mar;113(3):320-330. | Type of intervention (school-based interventions) |
| ‍‍16 | ‍Melendez-Torres GJ, Orr N, Farmer C, Shaw N, Chollet A, Rizzo AJ, Kiff F, Rigby E, Hagell A, Priolo Filho SR, Taylor B, Young H, Bonell C, Berry V. School-based interventions TO Prevent Dating and Relationship Violence and Gender-Based Violence: STOP-DRV-GBV systematic review. *Public Health Res (Southampt).* 2024 Feb;12(3):1-192. | Type of intervention (school-based interventions) |
| 17 | ‍Kiani Z, Simbar M, Rashidi Fakari F, Kazemi S, Ghasemi V, Azimi N, Mokhtariyan T, Bazzazian S. A systematic review: Empowerment interventions to reduce domestic violence? *Aggression and Violent Behavior*. 2021;58:101585. | Few primary studies were conducted in healthcare settings. |
| ‍‍18 | ‍Novitzky P, Janssen J, Kokkeler B. A systematic review of ethical challenges and opportunities of addressing domestic violence with AI-technologies and online tools. *Heliyon*. 2023 Jun 10;9(6):e17140. | Few primary studies were conducted in healthcare settings. |
| 19 | ‍Craven LC, Fields AM, Carlson RG, Combs EM, Howe ES. Counseling interventions for victims of intimate partner violence: a systematic review*. J Couns Dev.* 2023;101(3):346-58. | Few primary studies were conducted in healthcare settings. |
| ‍‍20 | ‍Kirk L, Terry S, Lokuge K, Watterson JL. Effectiveness of secondary and tertiary prevention for violence against women in low and low-middle income countries: a systematic review. *BMC Public Health.* 2017 Jul 4;17(1):622. | Few primary studies were conducted in healthcare settings. |
| 21 | ‍Sabri B, Sellke R, Smudde M, Bourey C, Murray SM. Gender-Based Violence Interventions in Low- and Middle-Income Countries: A Systematic Review of Interventions at Structural, Community, Interpersonal, Individual, and Multiple Levels. *Trauma Violence Abuse.* 2023 Dec;24(5):3170-3186. | Few primary studies were conducted in healthcare settings. |
| ‍‍22 | ‍Awolaran O, Olubumuyi O, OlaOlorun F, Assink M, van Rooij F, Leijten P. Interventions to reduce intimate partner violence against women in low- and middle-income countries: a meta-analysis. *Aggress Violent Behav.* 2022;64:101746. | Few primary studies were conducted in healthcare settings. |
| 23 | ‍Sabri B, Tharmarajah S, Njie-Carr VPS, Messing JT, Loerzel E, Arscott J, Campbell JC. Safety Planning With Marginalized Survivors of Intimate Partner Violence: Challenges of Conducting Safety Planning Intervention Research With Marginalized Women. *Trauma Violence Abuse.* 2022 Dec;23(5):1728-1751. | Few primary studies were conducted in healthcare settings. |
| ‍‍24 | ‍Babaee E, Tehrani-Banihashem A, Asadi-Aliabadi M, Sheykholeslami A, Purabdollah M, Ashari A, Nojomi M. Population-Based Approaches to Prevent Domestic Violence against Women Using a Systematic Review. *Iran J Psychiatry.* 2021 Jan;16(1):94-105. | Few primary studies were conducted in healthcare settings. |
| 25 | ‍Cork C, White R, Noel P, Bergin N. Randomized Controlled Trials of Interventions Addressing Intimate Partner Violence in Sub-Saharan Africa: A Systematic Review. *Trauma Violence Abuse.* 2020 Oct;21(4):643-659. | Few primary studies were conducted in healthcare settings. |
| ‍‍26 | ‍Owens J, Aboul-Enein BH, Bernstein J, Dodge E, J Kelly P. Reducing Violence Against Women and Girls in the Arab League: A Systematic Review of Preventive Interventions. *Trauma Violence Abuse.* 2024 Jul;25(3):2219-2233. | Few primary studies were conducted in healthcare settings. |
| 27 | Keith T, Hyslop F, Richmond R. A Systematic Review of Interventions to Reduce Gender-Based Violence Among Women and Girls in Sub-Saharan Africa*. Trauma Violence Abuse.* 2023 Jul;24(3):1443-1464. | Few primary studies were conducted in healthcare settings. |
| ‍‍28 | ‍Devine A, Spencer A, Eldridge S, Norman R, Feder G. Cost-effectiveness of Identification and Referral to Improve Safety (IRIS), a domestic violence training and support programme for primary care: a modelling study based on a randomised controlled trial. *BMJ Open.* 2012 Jun 22;2(3):e001008. | Type of study (RCT) |
| 29 | ‍Saboury Yazdy N, Talaei A, Ebrahimi M, Ghofrani Ivari A, Pouriran MA, Faridhosseini F, Mohaddes Ardabili H. "Be my Voice" to break social stigma against domestic violence: The underestimated role of smartphone applications in protecting victims in developing countries. *Front Psychiatry.* 2022 Aug 4;13:954602. | Type of study (opinion article) |
| ‍‍30 | ‍Rivas C, Vigurs C, Cameron J, Yeo L. A realist review of which advocacy interventions work for which abused women under what circumstances. *Cochrane Database Syst Rev*. 2019 Jun 29;6(6):CD013135. | Type of study (realist review) |
| 31 | ‍Philbrick W, Milnor J, Deshmukh M, Mechael P. Information and communications technology use to prevent and respond to sexual and gender-based violence in low- and middle-income countries: An evidence and gap map. *Campbell Syst Rev*. 2022 Oct 25;18(4):e1277. | Type of study (evidence and gap map) |
| ‍‍32 | ‍Weeks LE, Stilwell C, Rothfus M, Weeks AJ, Macdonald M, Jackson LA, Dupuis-Blanchard S, Carson A, Moody E, Helpard H, Daclan A. A Review of Intimate Partner Violence Interventions Relevant to Women During the COVID-19 Pandemic. Violence Against Women. 2024 Mar;30(3-4):981-1021. | Type of study (umbrella review) |
| 33 | ‍ Boyle M, Murphy-Tighe S. An integrative review of community nurse-led interventions to identify and respond to domestic abuse in the postnatal period*. J Adv Nurs.* 2022 Jun;78(6):1601-1617. | Type of study (integrative review) |
| ‍‍34 | ‍O’Brien JE, Macy RJ. Culturally specific interventions for female survivors of gender-based violence. *Aggress Violent Behav*. 2016;31:48–60. | Type of study (comprehensive review) |
| 35 | ‍El-Bassel N, Mukherjee TI, Stoicescu C, Starbird LE, Stockman JK, Frye V, Gilbert L. Intertwined epidemics: progress, gaps, and opportunities to address intimate partner violence and HIV among key populations of women. Lancet HIV. 2022 Mar;9(3):e202-e213. | Type of study (review) |
| ‍‍36 | ‍Yount KM, Krause KH, Miedema SS. Preventing gender-based violence victimization in adolescent girls in lower-income countries: Systematic review of reviews. *Soc Sci Med*. 2017 Nov;192:1-13. doi: 10.1016/j.socscimed.2017.08.038. | Type of study (umbrella review) |
| 37 | ‍Godenzi A, De Puy J. Overcoming boundaries: a cross-cultural inventory of primary prevention programs against wife abuse and child abuse. *J Prim Prev*. 2001;21:455-75. | Type of study (qualitative overview ) |
| ‍‍38 | ‍Houry D, Kaslow NJ, Kemball RS, McNutt LA, Cerulli C, Straus H, Rosenberg E, Lu C, Rhodes KV. Does screening in the emergency department hurt or help victims of intimate partner violence? *Ann Emerg Med.* 2008 Apr;51(4):433-42, 442.e1-7. | Type of study (observational study) |
| 39 | ‍Wagman JA, Gray RH, Campbell JC, Thoma M, Ndyanabo A, Ssekasanvu J, Nalugoda F, Kagaayi J, Nakigozi G, Serwadda D, Brahmbhatt H. Effectiveness of an integrated intimate partner violence and HIV prevention intervention in Rakai, Uganda: analysis of an intervention in an existing cluster randomised cohort. *Lancet Glob Health.* 2015 Jan;3(1):e23-33. | Type of study (analysis of an intervention) |
| ‍‍40 | ‍Gilbert L, Raj A, Hien D, Stockman J, Terlikbayeva A, Wyatt G. Targeting the SAVA (Substance Abuse, Violence, and AIDS) Syndemic Among Women and Girls: A Global Review of Epidemiology and Integrated Interventions. *J Acquir Immune Defic Syndr.* 2015 Jun 1;69 Suppl 2(0 2):S118-27. | Type of study (global review) |
| 41 | ‍Menon B, Stoklosa H, Van Dommelen K, Awerbuch A, Caddell L, Roberts K, Potter J. Informing Human Trafficking Clinical Care Through Two Systematic Reviews on Sexual Assault and Intimate Partner Violence. *Trauma Violence Abuse*. 2020 Dec;21(5):932-945. | Study background |
| 42 | ‍Sabri B, Mani SS, Kaduluri VPS. Integrated domestic violence and reproductive health interventions in India: a systematic review. *Reprod Health.* 2024 Jun 29;21(1):94. | Study background |
| 43 | ‍Eisenhut K, Sauerborn E, García-Moreno C, Wild V. Mobile applications addressing violence against women: a systematic review*. BMJ Glob Health.* 2020 Apr 22;5(4):e001954. | Study background |
| 44 | ‍Román-Gálvez RM, Martín-Peláez S, Fernández-Félix BM, Zamora J, Khan KS, Bueno-Cavanillas A. Worldwide Prevalence of Intimate Partner Violence in Pregnancy. A Systematic Review and Meta-Analysis*. Front Public Health.* 2021 Aug 30;9:738459. | Study background |
| 45 | ‍Dai X, Chu X, Qi G, Yuan P, Zhou Y, Xiang H, Shi X. Worldwide Perinatal Intimate Partner Violence Prevalence and Risk Factors for Post-traumatic Stress Disorder in Women: A Systematic Review and Meta-analysis. *Trauma Violence Abuse.* 2024 Jul;25(3):2363-2376. | Study background |
| 46 | ‍Nyberg A, Kecklund G, Hanson LM, Rajaleid K. Workplace violence and health in human service industries: a systematic review of prospective and longitudinal studies*. Occup Environ Med.* 2021 Feb;78(2):69-81. | Study background |
| 47 | ‍MacGregor JCD, Naeemzadah N, Oliver CL, Javan T, MacQuarrie BJ, Wathen CN. Women's Experiences of the Intersections of Work and Intimate Partner Violence: A Review of Qualitative Research. *Trauma Violence Abuse*. 2022 Jan;23(1):224-240. | Study background |
| 48 | ‍Bair-Merritt MH, Crowne SS, Thompson DA, Sibinga E, Trent M, Campbell J. Why do women use intimate partner violence? A systematic review of women's motivations. *Trauma Violence Abuse.* 2010 Oct;11(4):178-89. | Study background |
| ‍‍49 | ‍Park S, Kim SH. Who Are the Victims and Who Are the Perpetrators in Dating Violence? Sharing the Role of Victim and Perpetrator. *Trauma Violence Abuse.* 2019 Dec;20(5):732-741. | Study background |
| ‍‍50 | ‍Meyer SR, Lasater ME, García-Moreno C. Violence against older women: A systematic review of qualitative literature. *PLoS One*. 2020 Sep 24;15(9):e0239560. | Population studied (inclusion of interventions involving older women) |
| ‍‍51 | ‍Sawyer S, Coles J, Williams A, Williams B. A systematic review of intimate partner violence educational interventions delivered to allied health care practitioners. *Med Educ*. 2016 Nov;50(11):1107-1121. | Population studied (inclusion of interventions involving healthcare practitioners) |
| ‍‍52 | ‍Alvarez CP, Davidson PM, Fleming C, Glass NE. Elements of Effective Interventions for Addressing Intimate Partner Violence in Latina Women: A Systematic Review. *PLoS One.* 2016 Aug 9;11(8):e0160518. | Population studied (inclusion of interventions involving couples) |
| ‍‍53 | ‍Ryan J, Roman NV. Family-centered interventions for intimate partner violence: a systematic review. *African Safety Promotion*. 2019;17(1):32-48. | Population studied (inclusion of interventions involving families and couples) |

**Table S4.** Implications for healthcare stakeholders, public policies and research

| **Evidence-based Interventions** | **Healthcare professionals** | **Healthcare managers and policy-makers** | **Public policies** | **Research** |
| --- | --- | --- | --- | --- |
| -First-line support interventions;  -Interventions to increase GBV detection and continuity of care, to break the cycle of violence, safety behaviors and mitigation of health consequences;  -Interventions aimed to empowering women throughout advocacy and counselling;  -Multicomponent interventions based on comprehensive integrated program;  -Expansion of existing platforms of digital health interventions incorporate recent advances in technology (e.g. electronic health records, recommendation sheet translated for multicultural groups)  **Frameworks**  -Culturally sensitive, promoting equity and respecting diversity (gender, ethnicity, race, language, cultural backgrounds)  -Woman-centred, systemic and intergenerational approach  -Use of validated approaches that fundament the interventions (e.g. Ecological model, Theory of Change);  -Incorporating educative actions and mental health as core component of the interventions to empowering women, community awareness, and to assist women in restoring their mental strength and well-being. | -Non-judgmental, empathic attitudes respecting the woman's autonomy and needs;  -Validation of the women's experiences and feelings;  -Ensuring safety;  -Providing suitable information and facilitate easy access to referrals. | -Development of guidelines, training, and tools to address GBV in key settings like those entry point to the treatment of injuries (e.g. Emergency Departments), reproductive healthcare services (e.g. ANC), and facilities within women's communities addressing vulnerable groups.  -Ensure suitable conditions to implement the GBV interventions for healthcare professionals such as:   - Institutional protocol that orients a standard operating procedure in healthcare setting; - Internal and external system for referral; - Ensure private environments, confidentiality and time to the screening and appropriate disclosure of sensitive topics.   -Supervision and support of providers preventing vicarious trauma and burnout;  -Ongoing training of health and non-health workers to increase knowledge and skills in screening and managing GBV cases;  -Clarity of the roles and responsibilities of healthcare professionals and managers to addressing GBV;  -Community engagement and actions that strength external network support collaboration. | -Financing and resource allocation for GBV programs and services;  -Multisectoral collaboration (health, justice, social services, education);  -Defining priorities, planning, implementing, monitoring and evaluation of GBV interventions engaging diverse stakeholders;  -Strength health information systems to increase surveillance, monitoring and evaluation;  -Strength national and local legislation to address GBV | -Studies to explore whether the rate of IPV disclosure and interventions effectiveness varies depending on the clinical setting, target populations and the method used to administer screening tools;  -Further trials to test the benefits of different strategies to screening in diverse settings and screening combined with advocacy or other interventions by healthcare professionals;  -Incorporation of cost-effectiveness analysis as part of the evaluation of the implementation outcomes;  -Evaluations of effectiveness of technology-based interventions in LMICs;  -Evaluation of benefices, potential harms and overall effects on morbidity and mortality of screening and GBV interventions, particularly among vulnerable groups (e.g. LGBTQ+ individuals);  -Evaluation how multifaceted interventions can be strengthened to reduce IPV while achieving improvement in other risk behaviors;  -Development of interventions theoretical frameworks;  Evaluation of implementation outcomes such as reach, effectiveness, acceptability, sustainability, feasibility among others core components derived from robust implementation-oriented frameworks;  -Evidence synthesis that contributes to inform public policies about GBV and the implementation of evidence-based interventions;  -Longitudinal studies to investigate the effectiveness of the interventions for the long-term;  -Larger-scale, high-quality research to determine whether intervention programs prevent or reduce domestic violence episodes or have any effect on health particularly among vulnerable groups (e.g. pregnant, migrant women);  -Expanding the research on the effectiveness and uptake of IPV screening programs for men and nonbinary patients and tools to support the identification of diverse forms of GBV (e.g. modern slavery);  -Research to understand the implications of the patient-provider relationship in disclosure or IPV; including attitudes and preferences of providers and users in the implementation of interventions;  -Expanding the research to address safety, equity, and the unintended consequences of the use of ICT, their uses to prevent IPV and to address post-IPV challenges, as well as the use of ICT tools to address IPV for women with disabilities, and the accessibility of these tools;  -Global collaboration to improve the clarity about key concepts, measurements, duration of follow-up, individual, organization and system- level outcomes on the pathway from intervention to GBV prevention. |
